# FABP5 coordinates lipid signaling that promotes prostate cancer metastasis

**DOI:** 10.1038/s41598-019-55418-x

**Published:** 2019-12-12

**Authors:** Gregory Carbonetti, Tessa Wilpshaar, Jessie Kroonen, Keith Studholme, Cynthia Converso, Simon d’Oelsnitz, Martin Kaczocha

**Affiliations:** 1 Department of Biochemistry and Cell Biology, Stony Brook University, Stony Brook, NY 11794 USA; 2 Department of Anesthesiology, Stony Brook University, Stony Brook, NY 11794 USA; 3 Graduate Program in Molecular and Cellular Biology, Stony Brook University, Stony Brook, NY 11794 USA; 4 Institute of Chemical Biology and Drug Discovery, Stony Brook University, Stony Brook, NY 11794 USA

**Keywords:** Cell invasion, Prostate cancer, Metastasis

## Abstract

Prostate cancer (PCa) is defined by dysregulated lipid signaling and is characterized by upregulation of lipid metabolism-related genes including fatty acid binding protein 5 (FABP5), fatty acid synthase (FASN), and monoacylglycerol lipase (MAGL). FASN and MAGL are enzymes that generate cellular fatty acid pools while FABP5 is an intracellular chaperone that delivers fatty acids to nuclear receptors to enhance PCa metastasis. Since FABP5, FASN, and MAGL have been independently implicated in PCa progression, we hypothesized that FABP5 represents a central mechanism linking cytosolic lipid metabolism to pro-metastatic nuclear receptor signaling. Here, we show that the abilities of FASN and MAGL to promote nuclear receptor activation and PCa metastasis are critically dependent upon co-expression of FABP5 *in vitro* and *in vivo*. Our findings position FABP5 as a key driver of lipid-mediated metastasis and suggest that disruption of lipid signaling via FABP5 inhibition may constitute a new avenue to treat metastatic PCa.

## Introduction

Prostate cancer (PCa) remains the second leading cause of cancer-related death in men in the United States. Dysregulated lipid metabolism and signaling are prominent features of PCa^1–3^. Prostate tumors rely heavily upon endogenously synthesized and exogenously acquired lipids to fuel their growth, and disrupting lipid biosynthesis and/or uptake holds therapeutic promise in treating PCa^1,2,4,5^. Numerous lipid-metabolizing enzymes have been implicated as drivers of metastatic PCa. Prominent among these is fatty acid synthase (FASN), the enzyme that synthesizes *de novo* fatty acids and whose upregulation defines a subtype of PCa^2,3^. FASN expression is elevated in metastatic PCa and pharmacological FASN inhibition induces cellular cytotoxicity to limit tumor growth^2^. Monoacylglycerol lipase (MAGL) is an enzyme that hydrolyzes 2-monoacylglycerols into fatty acids and likewise promotes the growth of prostate tumors^6,7^.

Lipids generated by FASN and MAGL play key roles in the survival and growth of prostate tumors but may additionally engage signaling networks that promote metastasis. Peroxisome proliferator-activated receptor gamma (PPARγ) is a nuclear receptor that regulates the expression of pro-angiogenic genes that enhance metastasis, and its expression in metastatic PCa inversely correlates with patient survival^1,8,9^. In addition to PPARγ, other receptors including the related PPAR_β/δ_ as well as estrogen-related receptor α are known to increase the metastatic potential of PCa^10–14^. Thus, numerous redundancies exist in cytosolic lipid-metabolizing enzymes and nuclear receptors that promote PCa metastasis, and disrupting multiple components of lipid metabolism and/or signaling may be required to effectively attenuate tumor growth and metastasis^4^.

Signaling lipids generated by FASN and MAGL must gain entry to the nucleus to engage receptors, including PPARγ. Fatty acid binding proteins (FABPs) are a class of intracellular lipid chaperones that bind to and facilitate the transport of long-chain fatty acids and related lipids to various cellular compartments, including the nucleus^15^. Humans express ten distinct FABP isoforms. FABP5 is not expressed in the normal prostate but becomes highly upregulated in advanced metastatic PCa^16–22^. A similar pattern is evident in PCa cell-lines, wherein FABP5 expression is low or absent in weakly metastatic cell-lines and highest in the most aggressive and metastatic cell-lines^9,10,23–25^. Importantly, FABP5 overexpression enhances tumor growth and metastasis while pharmacological or genetic FABP5 inhibition suppresses PCa metastasis^18,26^. Mechanistically, the pro-metastatic effects of FABP5 are mediated by activation of PPARγ and estrogen-related receptor α^10,23^.

FABP5 thus represents a key transport protein delivering cytosolic lipids to nuclear receptors to promote a metastatic PCa phenotype. Given the robust increase in fatty acid metabolism and upregulation of FABP5 in metastatic PCa, we hypothesized that FABP5 may represent a central mechanism linking cytosolic lipid biosynthesis to pro-metastatic nuclear signaling. Here, using FASN and MAGL as prototypical examples, we show that the ability of these lipid-metabolizing enzymes to enhance PCa metastasis *in vitro* and *in vivo* is critically dependent upon FABP5, thus positioning FABP5 as a key node in a lipid signaling network that promotes PCa metastasis.

## Results

### FASN and MAGL enhance the metastatic potential of PCa cells only in the presence of FABP5

**Figure 1:**
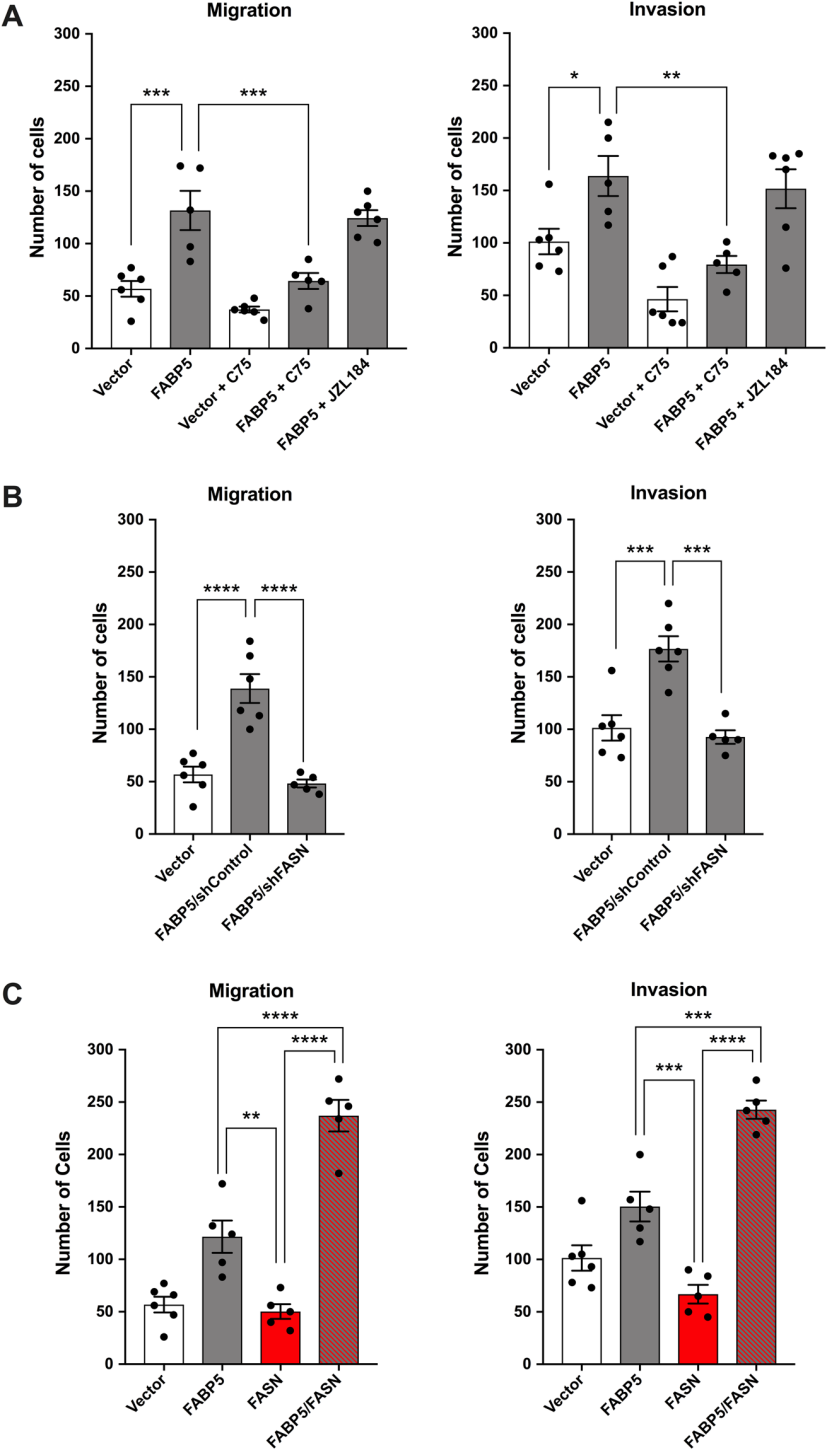
FASN enhances the metastatic potential of LNCaP cells only in the presence of FABP5. (**A**) Migration and invasion of LNCaP cells expressing FABP5 (grey bars) or vector control (white bars). The cells were treated with vehicle, the FASN inhibitor C75 (40 µM), or the MAGL inhibitor JZL184 (10 µM). (**B**) Effect of FASN knockdown upon migration and invasion of FABP5 expressing LNCaP cells. (**C**) Overexpression of FASN in LNCaP cells (red bars) increases their migration and invasion only in cells co-expressing FABP5 (red and grey bars). Data are presented as means ± SEM. *p < 0.05; **p < 0.01; ***p < 0.001; ****p < 0.0001; (n ≥ 5).

LNCaP cells are weakly metastatic and androgen-dependent. Overexpression of human FABP5 enhanced the migratory and invasive potential of LNCaP cells relative to empty-vector controls (Fig. 1A; Supplementary Fig. S1). FABP5 is a lipid chaperone and we reasoned that cytosolic enzymes such as FASN or MAGL provide FABP5 with a source of ligands to promote PCa metastasis. LNCaP cells robustly express FASN while MAGL is expressed at low, albeit detectable levels (Supplementary Fig. S1). To determine whether FASN activity is necessary for FABP5 to enhance the metastatic potential of LNCaP cells, we treated cells with the FASN inhibitor C75^27^, which does not appreciably inhibit FABP5 or adversely impact normal cellular proliferation over the time course of our studies (Supplementary Fig. S2). While C75 (40 µM) had no significant effect upon the migratory and invasive capacity of control LNCaP cells, it reduced migration and invasion of FABP5-expressing cells to a level comparable to vector alone (Fig. 1A). Similar effects were observed upon shRNA-mediated knockdown of FASN (Fig. 1B; Supplementary Fig. S1), indicating that FASN provides a source of lipids that enhance migration/invasion via FABP5. We next assessed whether FABP5 is similarly required for FASN to increase cellular migration and invasion. Overexpression of FASN in LNCaP cells failed to increase their metastatic potential compared to vector controls (Fig. 1C; Supplementary Fig. S1). In contrast, concomitant overexpression of FASN and FABP5 increased cellular migration and invasion (Fig. 1C; Supplementary Fig. S1) to a level greater than introduction of FABP5 alone. Collectively, these results indicate that FASN activity is required for FABP5 to enhance the metastatic potential of LNCaP cells and conversely that FABP5 is essential for FASN to increase cellular migration and invasion.

**Figure 2:**
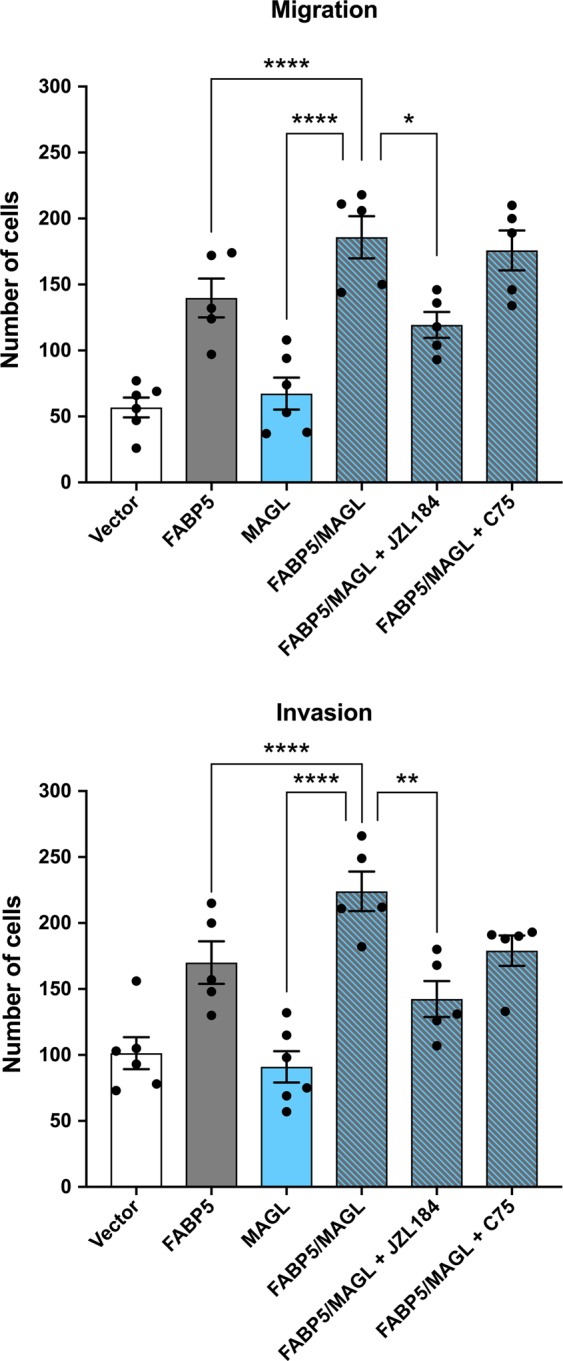
MAGL enhances the metastatic potential of LNCaP cells only in the presence of FABP5. Overexpression of MAGL in LNCaP cells (blue bars) increases migration and invasion only when FABP5 is co-expressed (blue and grey bars). Cells overexpressing FABP5 and MAGL were also treated with vehicle, C75 (40 µM), or JZL184 (10 µM). Data are presented as means ± SEM. *p < 0.05; **p < 0.01; ****p < 0.0001; (n ≥ 5).

We next assessed whether the ability of FABP5 to enhance the metastatic potential of PCa cells is unique to FASN-derived lipids or agnostic of the fatty acid origin. MAGL is an enzyme that cleaves 2-monoacylglycerols to generate free fatty acids. Similar to FASN, overexpression of MAGL in LNCaP cells had no effect upon their migratory and invasive potential (Fig. 2; Supplementary Fig. S1), consistent with previous work^7^. However, co-expression of MAGL and FABP5 increased cellular metastatic potential to an extent greater than that observed in cells overexpressing FABP5 alone (Fig. 2; Supplementary Fig. S1). To further explore the interplay between MAGL and FABP5, we treated LNCaP cells expressing FABP5 with the selective MAGL inhibitor JZL184^28^, which also did not negatively affect normal cellular proliferation (Supplementary Fig. S2). JZL184 (10 µM) had no effect upon the metastatic potential of FABP5 expressing cells (Fig. 1A), which we attribute to their robust expression of FASN (Supplementary Fig. S1). However, JZL184 reduced migration and invasion in LNCaP cells co-expressing MAGL and FABP5 (Fig. 2), confirming that the enhancement of their metastatic potential is dependent upon MAGL activity. In contrast, incubation of cells co-expressing MAGL and FABP5 with C75 did not significantly reduce migration or invasion (Fig. 2), indicating that MAGL-derived lipids are able to promote metastasis in the absence of FASN activity. Interestingly, in contrast to the reported changes in long chain free fatty acids observed upon acute FASN or MAGL inhibition^2,7,29^, we did not detect changes in fatty acids in our cell-lines (Supplementary Fig. S3). Taken together, our results demonstrate that FASN and MAGL enhance the metastatic potential of LNCaP cells only in the presence of FABP5.

**Figure 3:**
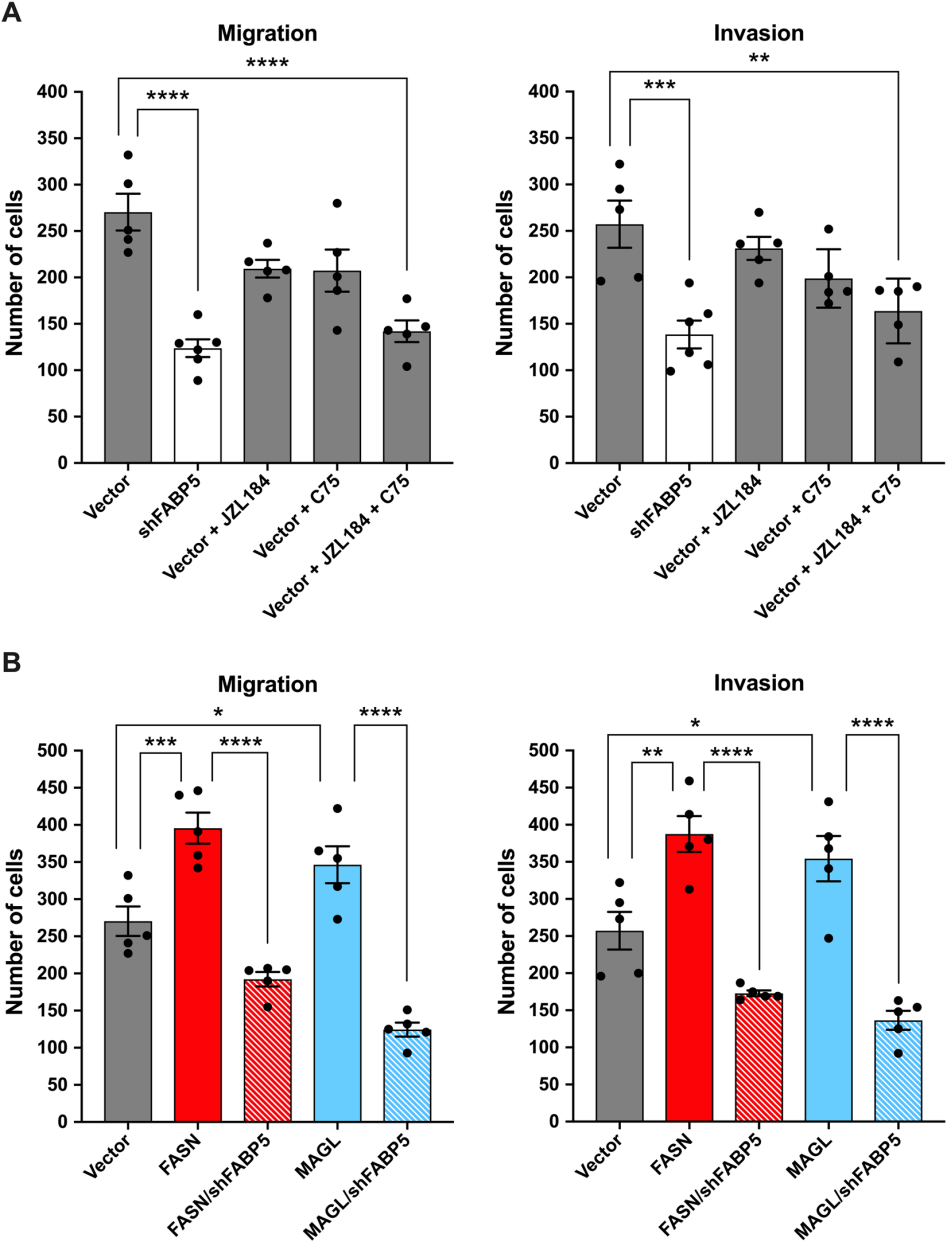
FABP5 knockdown suppresses metastatic potential in PC3 cells that express FASN and/or MAGL. (**A**) Migration and invasion of PC3 cells bearing an FABP5 knockdown (white bars) or vector control (grey bars), as well as migration and invasion of PC3 cells treated with vehicle, JZL184 (10 µM), C75 (40 µM), or a combination of both. (**B**) Overexpression of FASN (red bars) or MAGL (blue bars) increases the metastatic potential of PC3 cells in vector-expressing but not FABP5 shRNA-expressing cells (red/blue and white bars). Data are presented as means ± SEM. *p < 0.05; **p < 0.01; ***p < 0.001; ****p < 0.0001; (n ≥ 5).

We next assessed whether the interplay between FASN, MAGL, and FABP5 extends to the more aggressive PCa cell-line PC3, which expresses FABP5, FASN, and MAGL (Supplementary Fig. S1). FABP5 knockdown reduced migration and invasion in PC3 cells (Fig. 3A; Supplementary Fig. S1), and co-incubation of cells with both JZL184 and C75 reduced migration and invasion to a larger extent than observed with either inhibitor alone (without affecting normal cellular proliferation over the time course of the study) (Fig. 3A; Supplementary Fig. S2), indicating that lipid pools originating from FASN and MAGL both contribute to the metastatic potential of PC3 cells. Consistent with this notion, overexpression of FASN or MAGL increased the migratory and invasive potential of PC3 cells (Fig. 3B; Supplementary Fig. S1). However, this increase was attenuated upon simultaneous knockdown of FABP5 (Fig. 3B). These results confirm that similar to LNCaP cells, FASN and MAGL enhance the metastatic potential of PC3 cells only in the presence of FABP5.

### Nuclear translocation and PPARγ activation are essential to produce a metastatic phenotype

**Figure 4:**
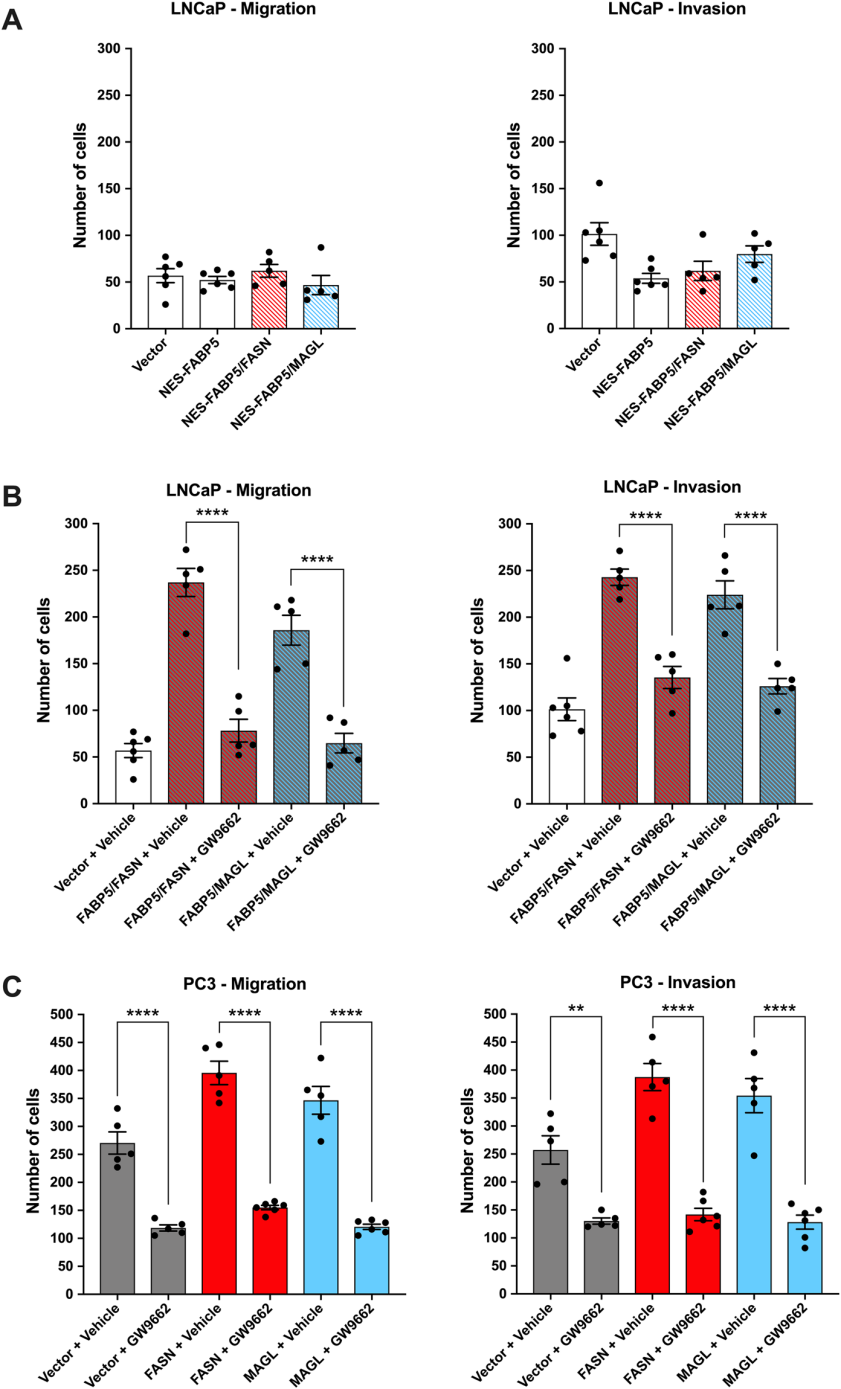
FABP5 transports MAGL- and FASN-derived lipids to PPARγ to produce a metastatic phenotype. (**A**) Migration and invasion of LNCaP cells co-expressing FASN or MAGL and NES-FABP5 (red/blue and white bars). (**B**) Migration and invasion of LNCaP cells co-expressing FABP5 and FASN or MAGL upon treatment with vehicle or the PPARγ antagonist GW9662 (10 µM). (**C**) The PPARγ antagonist GW9662 reduces migration and invasion in PC3 cells overexpressing FASN, MAGL, or vector control. Data are presented as means ± SEM. **p < 0.01; ****p < 0.0001; (n ≥ 5).

**Figure 5:**
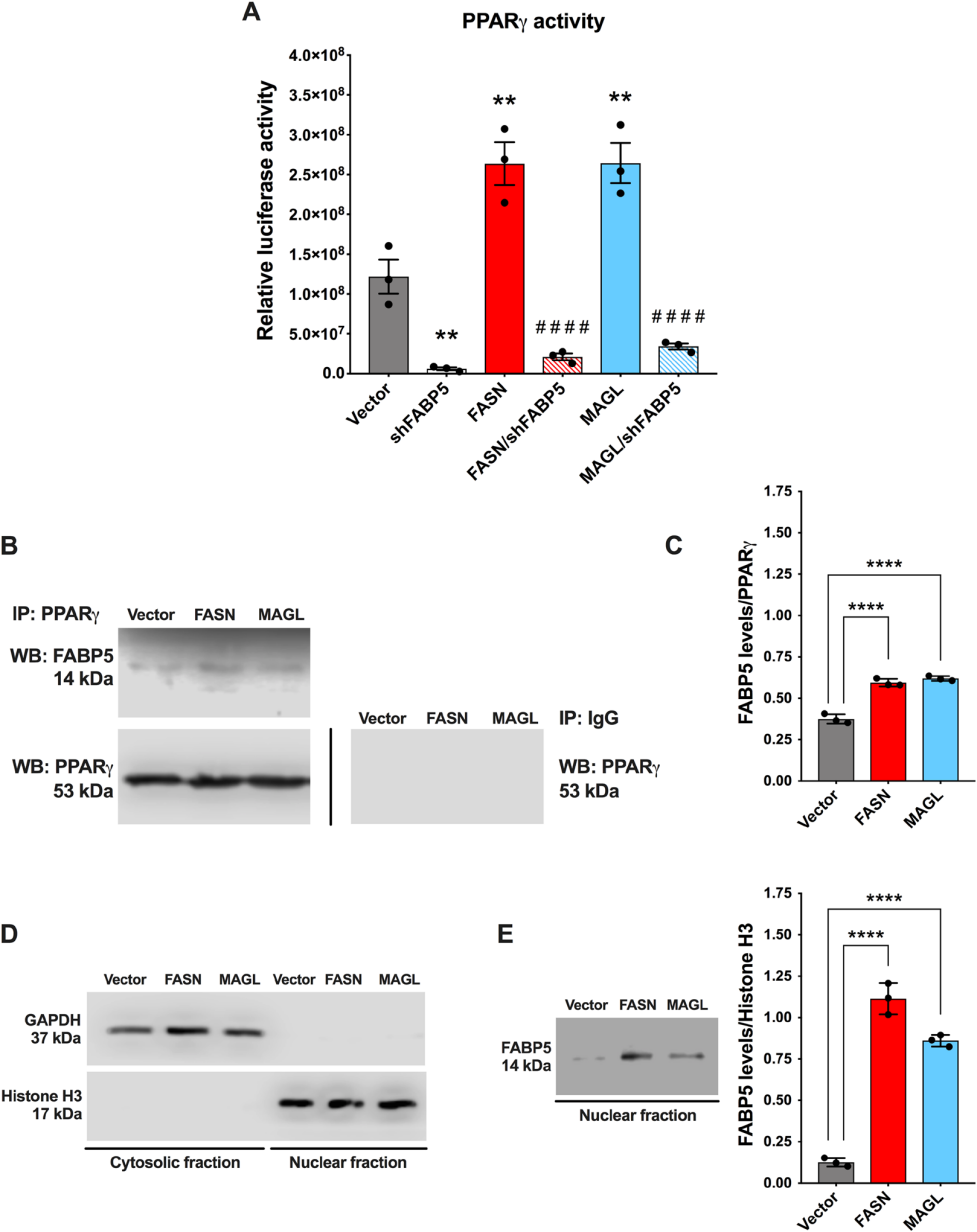
Knockdown of FABP5 lowers PPARγ activation, regardless of FASN and/or MAGL expression while increased interaction of PPARγ and FABP5 is observed when FASN/MAGL expression is elevated. (**A**) Relative PPARγ-luciferase activity (luciferase/β-galactosidase) in vector-expressing PC3 cells and PC3 cells bearing an FABP5 knockdown and/or overexpressing FASN or MAGL. Data are presented as means ± SEM. **p < 0.01 vs. vector. ^####^p < 0.0001 represents FASN vs. FASN/shFABP5 or MAGL vs. MAGL/shFABP5; (n = 3). (**B**) PPARγ immunoprecipitations (IP) were performed using PC3 vector control cells and PC3 cells overexpressing FASN or MAGL. IP samples contained antibody directed against PPARγ. The PPARγ IPs were western blotted using antibodies directed against FABP5 and PPARγ. The control antibody IPs (IgG1) were negative for FABP5. Samples were derived from the same experiment and blots were processed in parallel. (**C**) Following co-immunoprecipitation with PPARγ, FABP5 expression in vector-expressing PC3 cells and PC3 cells overexpressing FASN or MAGL was quantified using densitometry analysis of western blots. The signals were normalized to PPARγ. Data are presented as means ± SEM. ****p < 0.0001; (n = 3). (**D**) Western blot of vector-expressing PC3 cells and PC3 cells overexpressing FASN or MAGL following extraction of cytosolic and nuclear protein fractions. The purity of the collected fractions was corroborated using GAPDH and histone H3 as cytosolic and nuclear markers, respectively. Samples were derived from the same experiment and blots were processed in parallel. (**E**) *Left*, Western blot of FABP5 expression in the extracted nuclear fractions of PC3 vector control cells and PC3 cells overexpressing FASN or MAGL. *Right*, FABP5 expression was quantified using densitometry analysis of western blots. The signals were normalized to histone H3. Data are presented as means ± SEM. ****p < 0.0001; (n = 3).

Our results thus far indicate that FASN and MAGL produce lipids that converge upon FABP5 to enhance the metastatic potential of PCa cells *in vitro*. Given that cellular free fatty acid levels are not altered upon FABP5, FASN, and/or MAGL overexpression (Supplementary Fig. S3), we hypothesized that FABP5 promotes metastasis by regulating lipid signaling. Previous work has established that the nuclear receptor PPARγ mediates the pro-metastatic effects of FABP5 *in vitro*^8,23,30^. Thus, we assessed whether FASN and MAGL enhance the metastatic potential of PCa cells via PPARγ activation. We first generated an FABP5 variant that is excluded from the nucleus by fusing FABP5 with a nuclear export signal (NES) as we previously described (Supplementary Fig. S1; Supplementary Fig. S2)^31^. Expression of NES-FABP5 in LNCaP cells failed to increase migration and invasion even in cells simultaneously overexpressing MAGL or FASN (Fig. 4A). These results suggest that nuclear entry of FABP5 is critical to promote the metastatic potential of LNCaP cells. To determine whether activation of PPARγ mediates the effects of FABP5, FASN, and MAGL, we incubated LNCaP cells with the PPARγ antagonist GW9662 (10 µM). Treatment of cells co-expressing FABP5 and FASN or MAGL with GW9662 completely suppressed the metastatic potential of the cells to levels seen in vehicle treated LNCaP cells (Fig. 4B). Similar effects were observed in PC3 cells (Fig. 4C). We employed a PPARγ-luciferase system and confirmed that overexpression of FASN or MAGL significantly increased PPARγ activation while FABP5 knockdown attenuated PPARγ activity even in cells overexpressing FASN or MAGL (Fig. 5A). We next performed a co-immunoprecipitation and observed a direct interaction between PPARγ and FABP5 (Fig. 5B). Following densitometry analysis, there was significantly greater interaction between PPARγ and FABP5 in PC3 cells overexpressing FASN or MAGL compared to PC3 vector control cells (Fig. 5C). These findings were substantiated following the extraction of separate cytosolic and nuclear protein fractions (Fig. 5D) wherein PC3 cells overexpressing FASN or MAGL displayed greater accumulation of nuclear FABP5 relative to vector-expressing PC3 cells (Fig. 5E). Taken together, our results indicate that FABP5 mediates PPARγ activation upon FASN and MAGL overexpression to promote a metastatic phenotype.

### FABP5 is critical for FASN- and MAGL-mediated PCa metastasis *in vivo*

**Figure 6:**
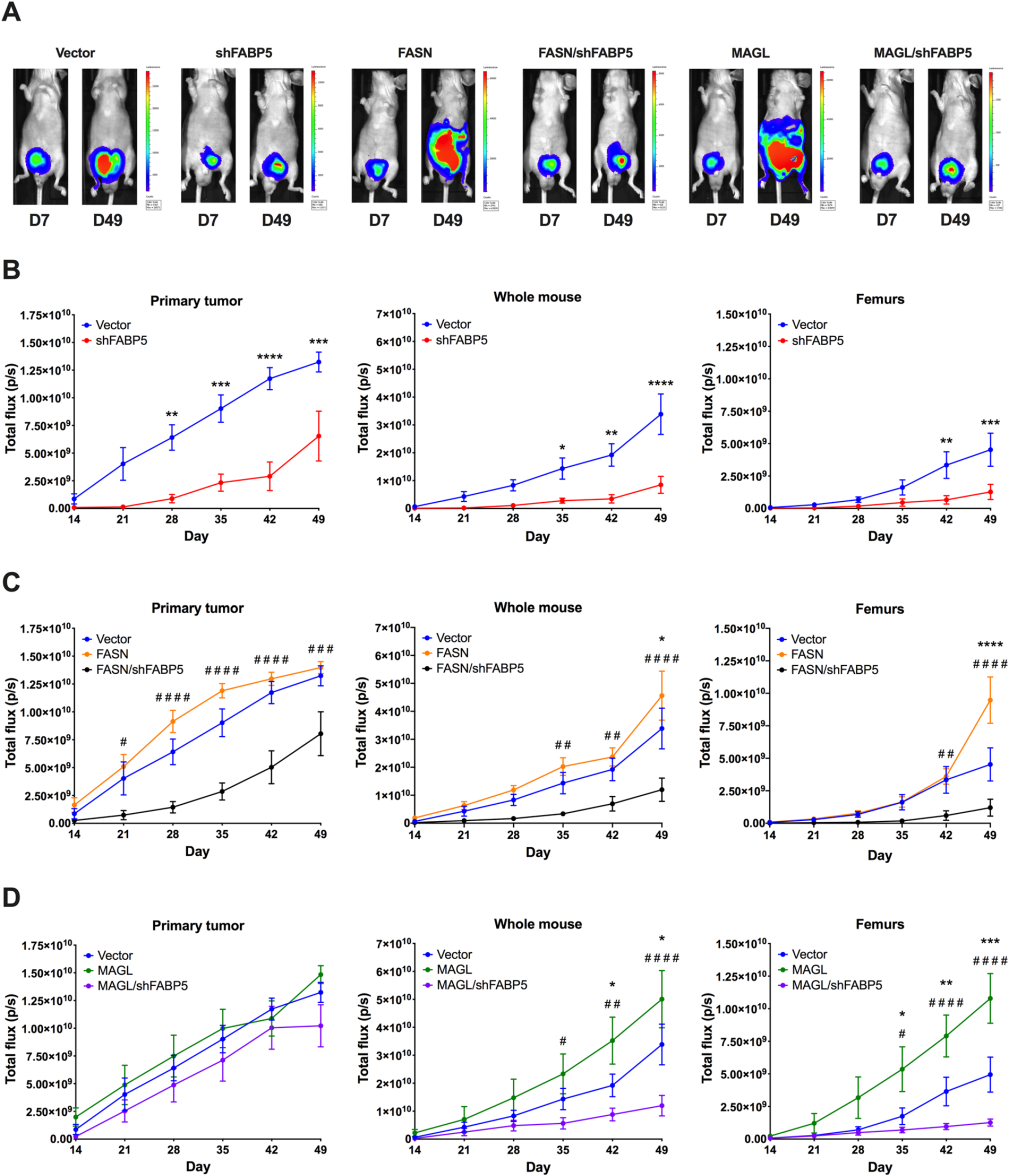
FABP5 is critical for FASN- and MAGL-mediated PCa metastasis *in vivo*. (**A**) Representative luciferase signals of PC3-Luc cells expressing vector, MAGL, or FASN on days 7 (D7) and 49 (D49). Signals are also shown for PC3-Luc cells expressing FABP5 shRNA (shFABP5) and those expressing FABP5 shRNA while overexpressing FASN (FASN/shFABP5) or MAGL (MAGL/shFABP5). (**B**) Total flux of vector and shFABP5 cells at the primary tumor site, whole mouse, and femurs. (**C**) Total flux of vector, FASN, and FASN/shFABP5 cells at the primary tumor site, whole mouse, and femurs. (**D**) Total flux of vector, MAGL, and MAGL/shFABP5 cells at the primary tumor site, whole mouse, and femurs. Data are presented as means ± SEM. *p < 0.05; **p < 0.01; ***p < 0.001; ****p < 0.0001 vs. vector. ^#^p < 0.05; ^##^p < 0.01; ^###^p < 0.001; ^####^p < 0.0001 represents FASN vs. FASN/shFABP5 or MAGL vs. MAGL/shFABP5; (n = 8).

**Figure 7:**
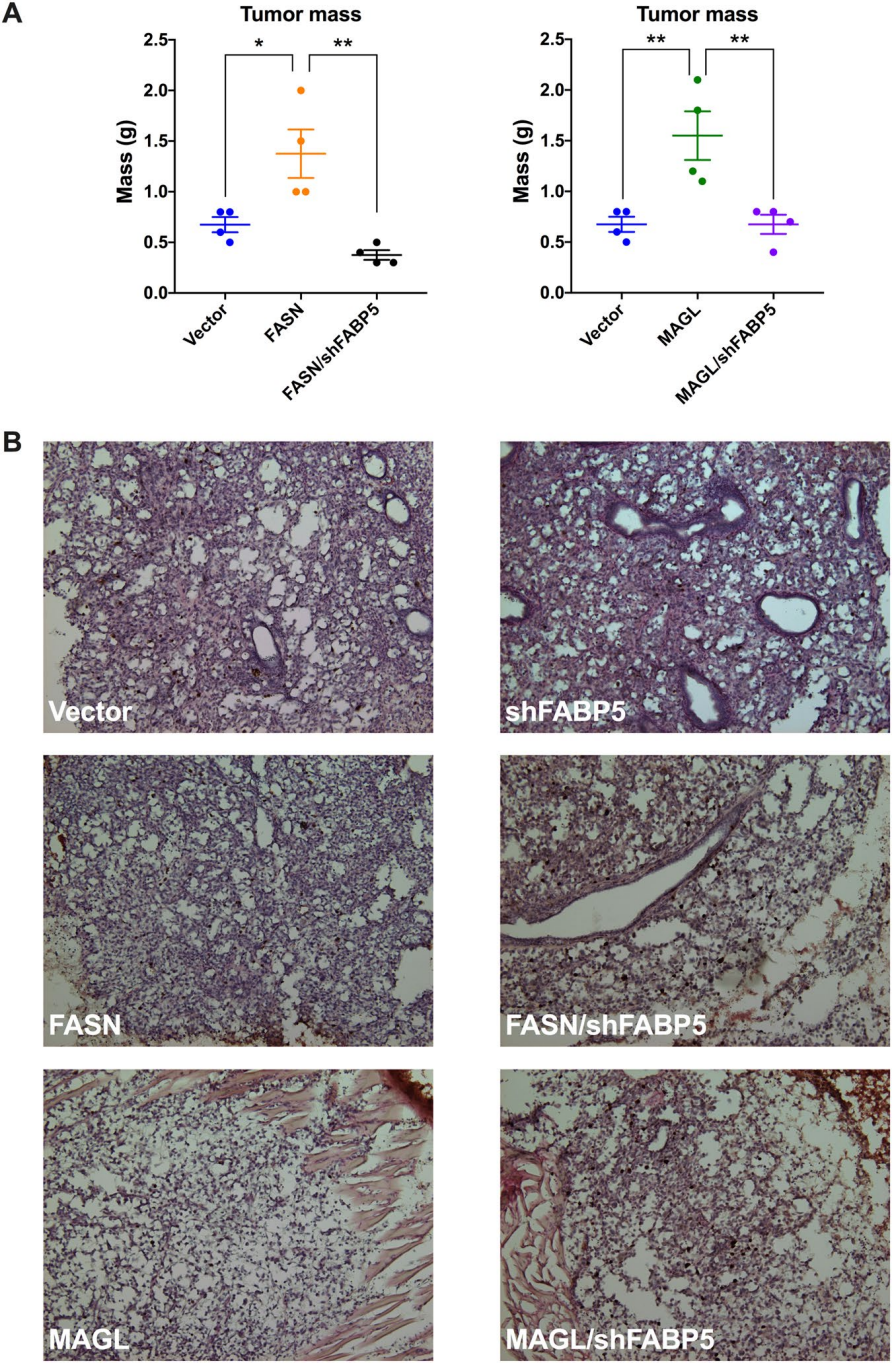
*Ex vivo* and histological analysis of primary tumor growth of mice orthotopically implanted with PC3-Luc cells. (**A**) Mass of excised vector, FASN, FASN/shFABP5, MAGL, and MAGL/shFABP5 primary tumors at 7 weeks post-implantation. Data are presented as means ± SEM. *p < 0.05; **p < 0.01; (n = 4). (**B**) Histological immunostaining on representative tumors from each animal cohort with hematoxylin and eosin (blue) and Ki-67 (brown): vector (top left), shFABP5 (top right), FASN (middle left), FASN/shFABP5 (middle right), MAGL (bottom left), and MAGL/shFABP5 (bottom right).

**Figure 8:**
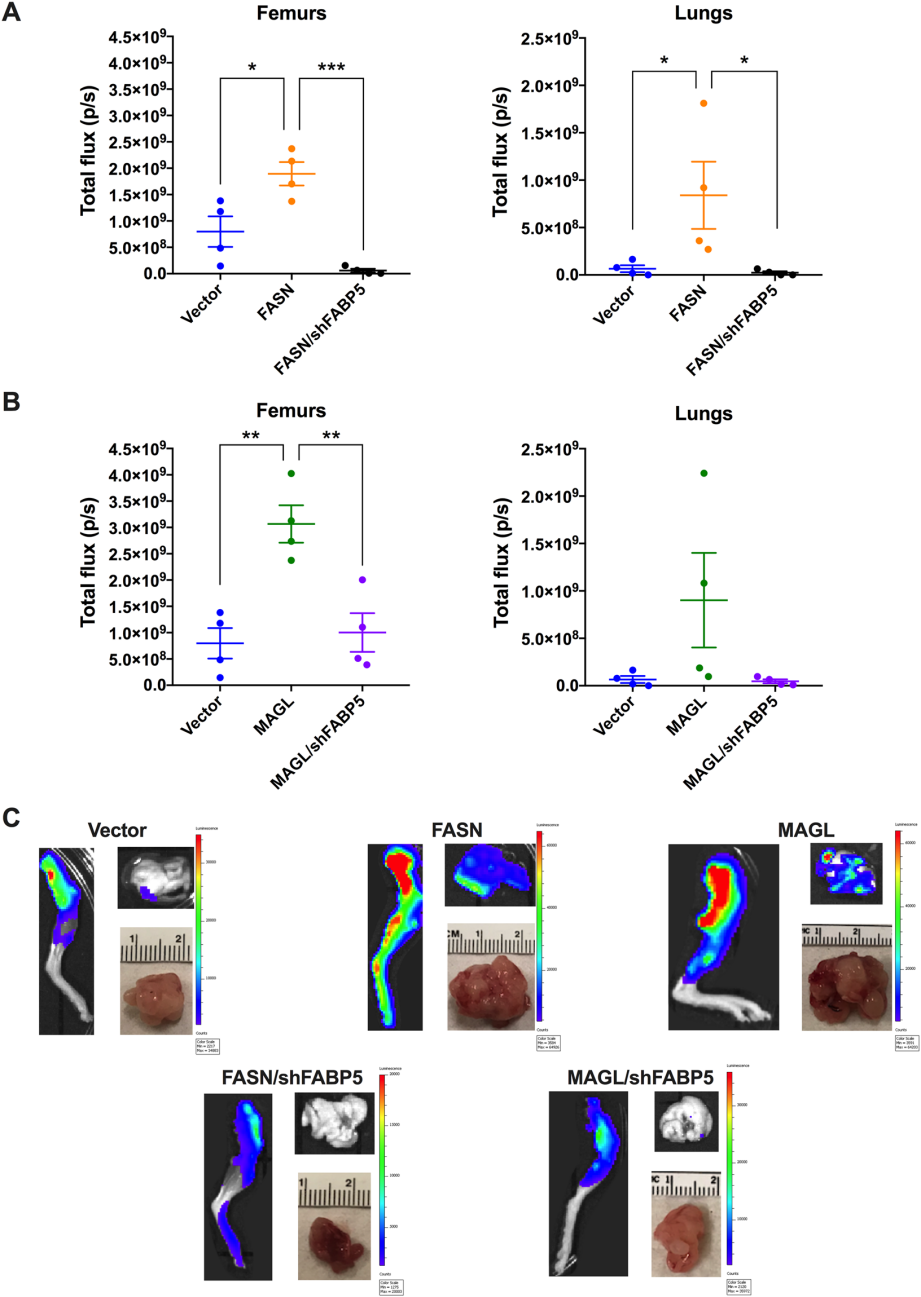
*Ex vivo* analysis of metastasis to femurs and lungs of mice orthotopically implanted with PC3-Luc cells. After week 7 post-implantation, femurs and lungs were analyzed. (**A**) Total flux of vector, FASN, and FASN/shFABP5 femurs and lungs. (**B**) Total flux of vector, MAGL, and MAGL/shFABP5 femurs and lungs. Data are presented as means ± SEM. *p < 0.05; **p < 0.01; ***p < 0.001; (n = 4). (**C**) Representative images of femurs (left), lungs (top right), and primary tumor (bottom right) taken from each cohort at the conclusion of week 7.

To determine whether FABP5 controls FASN and MAGL driven metastasis *in vivo*, we implanted PC3 cells expressing luciferase (PC3-Luc), whose migratory and invasive potential is comparable to PC3 cells, into the ventral lobe of the prostate gland of male BALB/c nude mice (Fig. 6A; Supplementary Fig. S2). We assessed the luciferase signal of the primary tumor, whole mouse, and metastatic sites (femurs and lungs) for up to 7 weeks after implantation *in vivo* (when mice began to display morbidity) (Fig. 6). At the conclusion of week 7, the tumors, femurs, and lungs of all extant mice were excised for *ex vivo* analysis. Tumors were weighed (Fig. 7A) and also subjected to histological immunostaining with Ki-67, which confirmed that the tumors of all experimental cohorts were still actively proliferating at the conclusion of experimentation (Fig. 7B)^32^. Luciferase signal in femurs and lungs was quantified *ex vivo* (Fig. 8).

Vector-expressing PC3-Luc cells (Vector) developed tumors that gradually metastasized to femurs while knockdown of FABP5 (shFABP5) reduced signal at the primary tumor, whole mouse, and femurs (Fig. 6B), confirming that FABP5 inhibition reduces tumor growth and metastasis. Compared to vector-expressing cells, overexpression of FASN (FASN) did not increase signal in the primary tumor *in vivo* but did lead to significantly higher signal in the whole mouse and femurs (Fig. 6C), indicating that FASN overexpression promotes metastasis of PC3-Luc cells. *Ex vivo* analysis confirmed higher total flux in the femurs and lungs of FASN-overexpressing cells at week 7 (Fig. 8A,C). In contrast to our *in vivo* findings, *ex vivo* analysis confirmed a larger tumor mass of FASN-overexpressing cells (Fig. 7A), indicating that FASN overexpression enhances tumor growth and metastasis. Compared to FASN-overexpressing cells, knockdown of FABP5 in FASN-overexpressing cells (FASN/shFABP5) significantly reduced luciferase signal in the primary tumor, whole mouse, and femurs *in vivo* (Fig. 6C), confirming that FABP5 knockdown suppresses the ability of FASN to promote metastasis. *Ex vivo* analysis of primary tumor mass and luciferase signal in femurs and lungs confirmed reduced tumor growth and metastasis in these FABP5 knockdown cells (Figs. 7A and 8A,C).

We next assessed whether FABP5 similarly controls the metastasis of MAGL-overexpressing cells (MAGL). MAGL overexpression in PC3-Luc cells significantly increased luciferase signal in the whole mouse and femur, which was confirmed *ex vivo* (Figs. 6D and 8B,C). Compared to MAGL-overexpressing cells, knockdown of FABP5 in cells overexpressing MAGL (MAGL/shFABP5) significantly reduced luciferase signal in the whole mouse and femurs (Fig. 6D), which was confirmed *ex vivo* (Fig. 8B,C). Similar to FASN-overexpressing cells, we did observe larger tumor mass of MAGL-overexpressing cells relative to vector-expressing cells *ex vivo*, which was suppressed upon FABP5 knockdown (Fig. 7A).

## Discussion

Converging evidence implicates dysregulated lipid metabolism and signaling as major drivers of PCa metastasis^1–3^. Accordingly, modulation of lipid metabolism holds therapeutic promise in the treatment of PCa, although the redundancy in lipid-metabolizing enzymes and nuclear receptors likely necessitates targeting multiple proteins in parallel. Our study provides evidence that FABP5 links cytosolic lipid metabolism and nuclear signaling, thus positioning FABP5 as a critical node in lipid signaling networks that drive PCa metastasis. Using FASN and MAGL as prototypical examples of fatty acid metabolizing enzymes commonly overexpressed in PCa, our study demonstrates that the pro-metastatic capabilities of these enzymes are critically dependent upon FABP5 expression *in vitro* and *in vivo*.

Using both the weakly metastatic LNCaP and the moderately metastatic PC3 cell-lines, we demonstrate that overexpression of FASN or MAGL in the absence of FABP5 fails to increase cellular migration and invasion. This is consistent with a previous report demonstrating that MAGL overexpression does not increase these parameters in LNCaP cells but does so in more aggressive PCa cell-lines^7^, which we now attribute to the lack of FABP5 expression in LNCaP cells. FABP5 overexpression enhances PCa metastasis^18,26^ and our results demonstrate that the ability of FABP5 to enhance metastasis is critically dependent upon FASN and/or MAGL activity. Notably, these effects are not dependent upon the activity of the androgen receptor (Supplementary Fig. S2). This interplay between FABP5, FASN, and MAGL extended to the *in vivo* setting as evidenced by suppression of metastasis in FABP5 knockdown cells despite the concomitant overexpression of FASN or MAGL.

FABP5 transports lipid ligands to nuclear receptors including PPARγ to promote metastasis^33,34^. Our results extend these findings by demonstrating that products of FASN, MAGL, and likely other lipid-metabolizing enzymes, rely upon FABP5 for PPARγ activation. From a therapeutic perspective, the noted redundancy in lipid-metabolizing enzymes and nuclear receptors suggests that targeting these proteins alone may not efficiently suppress metastasis. For example, many tumors weakly express FASN, while FABP5 also transports ligands to other nuclear receptors including PPAR_β/δ_ and estrogen-related receptor α^3,10,11,14^. It is noteworthy that dietary lipids enhance PCa progression and a high fat diet could subvert some of the beneficial effects exerted by blocking lipogenesis/lipolysis^35^. In addition to endogenously synthesized lipids, FABP5 translocates exogenous fatty acids to nuclear PPAR receptors^36,37^ and its inhibition would be expected to suppress metastasis induced by endogenously-synthesized as well as dietary lipids. Collectively, our study demonstrates that FABP5 plays a critical role in gating lipid-mediated metastasis and may represent a druggable node in a PCa lipid signaling network that drives metastasis.

## Methods

### Animal models

Male BALB/c nude mice (BALB/cOlaHsd-*Foxn1*^*nu*^, Envigo RMS Inc., Indianapolis, IN; RRID: MGI:2161064) were used for all *in vivo* experiments (20–30 g, 7–8 weeks old). Animals were housed at room temperature and kept on a 12:12-hour light:dark cycle with access to food and water *ad libitum*. Euthanasia was performed utilizing CO_2_ asphyxiation followed by decapitation. All of the experiments were approved by the Stony Brook University Institutional Animal Care and Use Committee (#850980).

### Cell-lines

Male LNCaP and PC3 cells were purchased from ATCC (ATCC, Manassas, VA; CRL-1740 and CRL-1435, RRID: CVCL_1379 and CVCL_0035, respectively) and authenticated by the ATCC human short-tandem repeat profiling cell authentication service (ATCC, Manassas, VA; STRA4168 and STRA4169, respectively). Male PC3-Red-FLuc cells (annotated as PC3-Luc) were purchased from PerkinElmer (PerkinElmer, Waltham, MA; BW128444, RRID: CVCL_5J08; Parental source: ATCC, Manassas, VA; CRL-1435). All three cell-lines (LNCaP, PC3, and PC3-Luc) were grown in RPMI 1640 (Gibco – Thermo Fisher Scientific, Gaithersburg, MD) supplemented with 10% fetal bovine serum (FBS) (Gemini Bio-Products, West Sacramento, CA) and 100 units/mL of penicillin/streptomycin (Gibco – Thermo Fisher Scientific, Gaithersburg, MD) in a humidified incubator set to 37 °C, containing 95% air and 5% CO_2_. Female HEK293T cells were purchased from ATCC (ATCC, Manassas, VA; CRL-3216, RRID: CVCL_0063). HEK293T cells were grown in Dulbecco’s Modified Eagle Medium (DMEM) (Gibco – Thermo Fisher Scientific, Gaithersburg, MD) supplemented with 10% FBS and 100 units/mL of penicillin/streptomycin in a humidified incubator set to 37 °C, containing 95% air and 5% CO_2_.

### Lentiviral constructs

Eight distinct lentiviral constructs were utilized to alter the protein expression levels of FABP5, FASN, and/or MAGL in LNCaP, PC3, and/or PC3-Luc cells. To overexpress human FABP5 in LNCaP cells, the pLenti-FABP5-Blast plasmid was established. Briefly, human FABP5 was amplified from the pET28a-hFABP5 plasmid (Kaczocha *et al*. 2012) using the following primers: 5′-CCGGATCCACCATGGCCACAGTTCAGCAG-3′ (forward) and 5′-AGTCTAGATTATTCTACTTTTTCATAGATCCGAGT-3′ (reverse)^31^. The amplicon was subcloned into the pLenti-CMV-Blast-empty plasmid (Addgene, Watertown, MA; Plasmid #: 17486; RRID: Addgene_17486) using *BamH1* and *Xba1* restriction enzymes. To drive the overexpression of FASN in LNCaP, PC3, and PC3-Luc cells, the pBK649-pLenti-FASN-Puro plasmid was constructed. Briefly, the retroviral plasmid pBabe-FASN-IRES-Luc, gifted by Dr. Massimo Loda (Dana-Farber Institute, Boston, MA), was sent to the Viral Vector Core at Duke University and the FASN open reading frame subcloned into a lentiviral plasmid (following excision of the IRES and luciferase domains)^38^. To drive overexpression of human MAGL in LNCaP, PC3, and PC3-Luc cells, the pLenti-MAGL-Puro plasmid was generated. Briefly, human MAGL was amplified from the pCDNA4-MGLL plasmid, gifted by Dr. Dale Deutsch (Stony Brook University, Stony Brook, NY), using the following primers: 5′-CCGGATCCACCATGGAAACAGGACCTGAAGACC-3′ (forward) and 5′-AGTCTAGATCAGTAATCTGGAACATCGTATGGGTAGGGTGGGGACGCAGTTCC-3′ (reverse)^39^. The amplicon was then subcloned into the pLenti-puro plasmid (Addgene, Watertown, MA; Plasmid #: 39481; RRID: Addgene_39481) using *BamH1* and *Xba1* restriction enzymes. To drive the expression of GFP-tagged human FABP5, the hFABP5-eGFP-N1 plasmid was established. Briefly, human FABP5 was amplified from the pET28a-hFABP5 plasmid using the following primers: 5′-GCCTCGAGGCCACAGTTCAGCAGCTG-3′ (forward) and 5′-GCGGTACCTTCTACTTTTTCATAGATCCGAGTACA-3′ (reverse). Next, mouse FABP5 was excised from the mFABP5-eGFP construct (via *Xho1* and *Kpn1* digestion), utilized by Kaczocha *et al*. 2012, and human FABP5 was subcloned in its place using *Xho1* and *Kpn1* restriction enzymes^31^. Similarly, to drive the expression of GFP-tagged human NES-FABP5, the NES-FABP5-eGFP-N1 plasmid was constructed. Briefly, human FABP5 was amplified from the pET28a-hFABP5 plasmid using the previously utilized primers: 5′-GCCTCGAGGCCACAGTTCAGCAGCTG-3′ (forward) and 5′-GCGGTACCTTCTACTTTTTCATAGATCCGAGTACA-3′ (reverse). Next, mouse FABP5 was excised from the NES-FABP5-eGFP-N1 construct (via *Xho1* and *Kpn1* digestion), utilized by Kaczocha *et al*. 2012, and human FABP5 was subcloned in its place using *Xho1* and *Kpn1* restriction enzymes^31^. To knockdown the expression of FABP5, a previously utilized lentiviral plasmid containing shRNA corresponding to human FABP5 was employed (shRNA clone V3LHS_402771; GE Dharmacon, Lafayette, CO)^31^. To knockdown the expression of FASN, a lentiviral plasmid containing shRNA corresponding to human FASN was purchased (shRNA clone V3LHS_332906; GE Dharmacon, Lafayette, CO). A non-silencing shRNA control for human FASN expression was also purchased (shRNA clone V3LHS_173006; GE Dharmacon, Lafayette, CO).

### Lentiviral packaging and transduction

All lentiviral plasmids utilized to alter protein expression levels in LNCaP, PC3, and PC3-Luc cells (pLenti-FABP5-Blast, pBK649-pLenti-FASN-Puro, pLenti-MAGL-Puro, hFABP5-eGFP-N1, NES-FABP5-eGFP-N1, V3LHS_402771, V3LHS_332906, and V3LHS_173006) were packaged into functional lentiviruses and used to infect host cells. HEK293T cells were sub-cultivated in a 10 cm cell culture plate to reach a confluency of 70–80% in complete DMEM (containing 10% FBS and 100 units/mL of penicillin/streptomycin). Next, the HEK293T cells were co-transfected with the desired lentiviral plasmid and the third-generation lentiviral packaging plasmids p-RSV-Rev (Addgene, Watertown, MA; Plasmid #: 12253, RRID: Addgene_12253), pCMV-VSV-G (Addgene, Watertown, MA; Plasmid #: 8454, RRID: Addgene_8454), and pCgpV (Cell Biolabs Inc., San Diego, CA; Plasmid #: 320024), at a 3:1:1:1 ratio using GenJet Plus transfection reagent (SignaGen, Rockville, MD) according to manufacturer’s instructions. After 24 hours, the media of transfected HEK293T cells was refreshed, and functional packaged lentiviruses were harvested from the media at 48 hours. LNCaP cells were infected at a multiplicity of infection (MOI) of 10, and PC3/PC3-Luc cells were infected at a MOI of 50. Twenty-four hours following transduction, cells were refreshed with complete media, and subsequently split at 48 hours. At 72 hours, cells were subjected to puromycin-selection (1 µg/mL) and/or blasticidin-selection (5 µg/mL) (MilliporeSigma, Burlington, MA). Appropriate overexpression/knockdown of proteins was assayed and confirmed via Western blotting. Infected cells were kept under selective pressure for at least one week and passaged at least once in the absence of selection prior to experimentation.

### Orthotopic implantation

Following lentiviral infection, PC3-Luc cells were propagated in culture and implanted orthotopically into the ventral lobe of the prostate gland of male BALB/c nude mice. Mice were anesthesized under 2.0% isoflurane anesthesia (Henry Schein, Melville, NY) and received continuous anesthesia throughout the entirety of the procedure. Additionally, mice received a subcutaneous injection of buprenorphine (0.1 mg/kg) (Henry Schein, Melville, NY). The surgical area (lower abdomen) was alternately swabbed 3 times with 70% ethanol and betadine. A low midline abdominal incision of 3–4 mm was then made utilizing sterile surgical scissors. Using sterile forceps, the bladder of the mice was lifted (without disturbing other organs or musculature) to expose the ventral lobe of the prostate gland found directly beneath the bladder (if necessary, fat was moved away using a sterile cotton swab). Using a 0.5 cc syringe with a 28 G needle, 2.5 × 10^5^ cells were resuspended in 20 µL of sterile PBS and implanted directly into the ventral lobe of the prostate gland. Following implantation, the bladder was replaced and the muscle layer closed using 4–0 absorbable vicryl monofilament sutures in an uninterrupted pattern. The skin layer was closed using sterile 9 mm staples. The animal was then removed from isoflurane anesthesia, placed on a heating pad during the recovery period, and monitored until awake and ambulatory. Mice were administered buprenorphine 4 hours post-surgery, followed by an additional administration every 12–24 hours for the next 48 hours. Staples were removed from the mice 7–10 days post-surgery once the incision wound had healed. Mice were continually monitored for weight and food consumption. Humane endpoints for all animals that underwent surgery were as follows: body weight decreasing by >15%, tumor/incision ulcerations, failure to groom, paralysis, respiratory distress, and/or bleeding. Sample size analysis was carried out using Russ Lenth’s Java Applets for Power and Sample Size computer software which determined 8 animals/group (one-way ANOVA with Tukey multiple comparisons test; SD = 20%; Power = 80%; Contrast difference detection = 30%).

### Animal imaging

Measurement of PC3-Luc cellular growth and metastasis was carried out utilizing a Caliper IVIS Spectrum imaging system (PerkinElmer, Waltham, MA). Beginning at 24 hours after orthotopic implantation, mice were imaged weekly for 7 weeks (when mice began to display morbidity). Briefly, mice received an intraperotineal injection of luciferin (150 mg/kg) (PerkinElmer, Waltham, MA) and were imaged 10 minutes later under 2.0% isoflurane anesthesia. At the conclusion of week 7, extant mice received a luciferin injection and femurs, lungs, and primary tumors were expediently excised, weighed, and imaged in a 10 cm cell culture plate. Luminescence was quantified as total flux (photons/second) using LivingImage software (PerkinElmer, Waltham, MA).

### Western blotting

20 µg of protein (or 50 µg of protein derived from co-immunoprecipitations) were run on a 10% sodium dodecyl sulfate/polyacrylamide gel electrophoresis (SDS/PAGE) gel at 90 V for 75 minutes. The protein was then transferred to a 0.45 µm nitrocellulose membrane (Bio-Rad, Hercules, CA) at 100 V for 25 minutes, followed by blocking in 5% non-fat dry milk in PBST. After blocking, the blots were trimmed and probed with mouse anti-β-actin (1:10,000) (Abcam, Cambridge, MA; Cat# ab6276, RRID: AB_2223210), rabbit anti-GAPDH (1:5000) (Cell Signaling Technology, Inc., Danvers, MA; Cat# 5174, RRID: AB_10622025), rabbit anti-histone H3 (1:2000) (Cell Signaling Technology, Inc., Danvers, MA; Cat# 4499, RRID: AB_10829039), sheep anti-FABP5 (1:1000) (BioVendor, Asheville, NC; Cat# RD1840060100, RRID: AB_1526706), rabbit anti-FABP5 (1:1000) (BioVendor, Asheville, NC; Cat# RD181060100, RRID: AB_344491), rabbit anti-FASN (1:500) (Abcam, Cambridge, MA; Cat# ab128856, RRID: AB_11143234), rabbit anti-MAGL (1:500) (Abcam, Cambridge, MA; Cat# ab124796, RRID: AB_10974580), mouse anti-PPARγ (1:1000) (Cell Signaling Technology, Inc., Danvers, MA; Cat# 95128, RRID: AB_2800240), or mouse anti-IgG1 (1:1000) (Cell Signaling Technology, Inc., Danvers, MA; Cat# 5415, RRID: AB_10829607) overnight with shaking at 4 °C. The blots were then washed at least three times with PBST, followed by probing with one of the three following HRP-conjugated secondary antibodies for 1 hour at room temperature: goat anti-rabbit IgG (1:5000) (Life Technologies - Thermo Fisher Scientific, Gaithersburg, MD; Cat# A16104, RRID: AB_2534776), goat anti-mouse IgG (1:5000) (Life Technologies - Thermo Fisher Scientific, Gaithersburg, MD; Cat# A16104, RRID: AB_2534745), or donkey anti-sheep IgG (1:15,000) (Jackson ImmunoResearch Laboratories Inc., West Grove, PA; Cat# 713-035-147, RRID: AB_2340710). The blots were then washed at least three times with PBST, followed by development using the Immun-star HRP substrate (Bio-Rad, Hercules, CA) and exposure to film. Densitometry analysis of western blots was carried out using ImageJ software (NIH, Bethesda, MD).

### Boyden chamber migration and invasion assays

Cells were pre-treated for 2 hours before the initiation of the experiment with C75 (40 µM) and/or JZL184 (10 µM), GW9662 (10 µM), bicalutamide (10 µM) (Cayman Chemical, Ann Arbor, MI), or vehicle (0.1% DMSO). Briefly, 210 µL of cell media (±the appropriate pharmacological agent(s)/vehicle) was added to the bottom well of a Neuro Probe blind well chemotaxis chamber (Neuro Probe Inc., Gaithersburg, MD), and a porous hydrophilic polycarbonate membrane (8.0 µm pore size, 13 mm diameter) was placed on top (MilliporeSigma, Burlington, MA). When carrying out invasion assays, 100 µL of Matrigel (300 ng/µL) (Corning Inc., Corning, NY) was added to the top of the polycarbonate membrane and allowed to solidify in a humidified incubator set to 37 °C for 2 hours. Cells to be utilized in the assay were then harvested, counted, resuspended in the appropriate culture media, and added to the top well of the chemotaxis chamber (50,000 cells in 200 µL of media per chamber (±the pharmacological agent(s)/vehicle)). The completed chemotaxis chamber was then placed in a humidified incubator set to 37 °C containing 95% air and 5% CO_2_ for 20 hours. After 20 hours, sterile cottons swabs were used on the top of the polycarbonate membrane to mechanically remove excess media, Matrigel (for invasion assays), and cells that had not migrated/invaded. Using forceps, the membrane was then removed from the chemotaxis chamber, placed in a 12-well plate, and the bottom fixed in 4% paraformaldehyde for 20 minutes at room temperature (Ted Pella Inc., Redding, CA). Following fixation, the paraformaldehyde was aspirated, and fixed cells were stained using Hoechst 33342 (1:1000) for 1 hour in the absence of light (Thermo Fisher Scientific, Gaithersburg, MD). After staining, the membrane was then mounted onto a slide, and all cells that had successfully migrated/invaded through the membrane were quantified using a Zeiss LSM 510 META NLO Two-Photon Laser Scanning Microscope (DAPI channel; 5x field of view) (Carl Zeiss Vision Inc., San Diego, CA).

### PPARγ transactivation assay

PC3 cells were seeded into 24-well plates to reach a confluency of 60–70%. Next, cells were co-transfected with plasmids encoding the PPARγ ligand binding domain fused to a GAL4 DNA binding domain (PPARγ LBD-GAL4-DBD), 4x upstream activation sequence for GAL4 upstream of luciferase (UAS 4x-TK Luc), and β-galactosidase as a transfection efficiency control (Kaczocha *et al*., 2012). Twenty-four hours later, the cells were lysed and processed using the Bright-Glo Luciferase Assay System (Promega Corporation, Madison, WI) and β-galactosidase Assay System (Promega Corporation, Madison, WI), according to manufacturer’s instructions. PPARγ activation (measured via luciferase luminescence at 595 nm) and β-galactosidase activity (measured via absorbance at 405 nm following hydrolysis of ο-nitrophenyl-β-d-galactopyranoside to ο-nitrophenyl) were quantified using an F5 Filtermax Multi-Mode Microplate Reader (Molecular Devices, Sunnyvale, CA). Background luminescence from non-transfected PC3 cells was subtracted from all samples, and PPARγ activity was reported as relative luciferase activity (luciferase/β-galactosidase).

### Cytoplasmic and nuclear protein extraction

Twenty-four hours prior to the initiation of extraction, PC3 vector control cells and PC3 cells overexpressing FASN or MAGL were sub-cultivated in 10 cm cell culture plates to reach a confluency of 70–80%. Cells were then harvested, and the cytosolic and nuclear protein fractions were isolated using the NE-PER Nuclear and Cytoplasmic Extraction Kit (Life Technologies - Thermo Fisher Scientific, Gaithersburg, MD) according to manufacturer’s instructions. Following extraction, the purity of the collected fractions was assessed via western blot using antibodies directed against GAPDH and histone H3 as cytosolic and nuclear markers, respectively.

### Proliferation assays

Vector expressing LNCaP or PC3 cells were seeded into 24-well plates (50,000 cells/well). Twenty-four hours later, JZL184 (10 µM) or C75 (40 µM) or JZL184 and C75 were added to the cells and subsequently incubated for 20 hours. Following incubation, cells were harvested and counted after the addition of trypan blue using a hemocytometer.

### Co-immunoprecipitation

PC3 vector control cells and PC3 cells overexpressing FASN or MAGL were harvested and lysed using ice-cold lysis buffer (20 mM Tris-HCl, 150 mM NaCl, 1 mM EDTA, 1% NP-40, pH 7.4) containing both protease and phosphatase inhibitors (MilliporeSigma, Burlington, MA). The cells were then scraped and centrifuged (13,000 g) for 15 minutes at 4 °C. Lysates were collected and incubated with 20 µL of 50% Protein G Agarose beads (Cell Signaling Technology, Inc., Danvers, MA; Cat# 37478) for 30 minutes at 4 °C on an end-over-end rocker. Lysates/beads were then centrifuged (13,000 g) for 10 minutes at 4 °C. Lysates were collected, diluted 1:2 in sterile PBS, and incubated with mouse anti-PPARγ (1:50) (Cell Signaling Technology, Inc., Danvers, MA; Cat# 95128, RRID: AB_2800240), or mouse anti-IgG1 (1:50) (Cell Signaling Technology, Inc., Danvers, MA; Cat# 5415, RRID: AB_10829607) antibodies overnight at 4 °C on an end-over-end rocker. Next, the lysates were incubated with 20 µL of 50% Protein G Agarose beads for 2 hours at 4 °C with gentle rocking. The samples were then centrifuged (13,000 g) for 30 seconds at 4 °C, and the supernatant discarded. The beads were then washed 3 times with 1 mL of sterile PBS before being boiled for 5 minutes and subjected to western blotting with antibodies directed against FABP5, PPARγ, and IgG1.

### Immunolocalization of FABP5

After LNCaP cells were transduced (as previously described) with either the hFABP5-eGFP-N1 or the NES-FABP5-eGFP-N1 plasmid, the cells were fixed using 4% paraformaldehyde (Ted Pella Inc., Redding, CA) for 15 minutes at room temperature and subsequently mounted onto slides using ProLong Gold Antifade Mountant (Thermo Fisher Scientific, Gaithersburg, MD). The images were captured using a Zeiss LSM 510 META NLO Two-Photon Laser Scanning Microscope (Carl Zeiss Vision Inc., San Diego, CA) using both the GFP-channel and DAPI-channel, and subsequent images were merged using AxioVision Software (Carl Zeiss Vision Inc., San Diego, CA).

### FABP5 protein purification

Human FABP5 was purified exactly as previously described^31^.

### Fluorescence displacement assay

Using 96-well plates, purified FABP5 (3 µM) was incubated with 0.5 µM NBD-stearate (Avanti Polar Lipids, Alabaster, AL) in 30 mM Tris-HCl, 100 mM NaCl buffer (pH 7.4) in the presence of positive control arachidonic acid (10 µM) (Cayman Chemical, Ann Arbor, MI) or increasing concentrations of the FASN inhibitor C75 (5–500 µM) (Cayman Chemical, Ann Arbor, MI). Loss of fluorescence intensity (attributable to NBD-stearate ligand displacement) was measured at the respective excitation and emission wavelengths of 466 nm and 520–566 nm using an F5 Filtermax Multi-Mode Microplate Reader (Molecular Devices, Sunnyvale, CA). Fluorescence of wells lacking FABP5 was subtracted from all samples.

### LC/MS

Palmitate (PA), oleate (OA), and stearate (SA) levels were quantified using liquid chromatography/mass spectrometry. Briefly, LNCaP and PC3 cells (5.0 × 10^5^) were harvested and homogenized in 4 mL of 2:1:1 chloroform:methanol:Tris (50 mM, pH 8) in the presence of 50 ng *d*_2_-PA and 50 ng *d*_17_-OA (Cayman Chemical, Ann Arbor, MI). Following centrifugation at 2800 RPM for 10 minutes at room temperature, the organic layer was dried down with argon and resuspended in 120 µL of 40% acetonitrile in water and subsequently injected into a Thermo TSQ Quantum Access Max mass spectrometer (Thermo Fisher Scientific, Gaithersburg, MD). LC separation was achieved on a Gemini C18 (50 × 2 mm) (Phenomenex, Torrance, CA). Mobile phase A consisted of H_2_O while mobile phase B was composed of acetonitrile and quantification was performed in the negative ion mode with select ion monitoring and the voltage set to 4 kV. The sheath pressure was 30 and the capillary was set to 270 °C. The flow rate was 200 µL/min. The gradient started at 40% B for 5 minutes, increased to 90% B over 10 minutes, was held at 90% B for 20 minutes, decreased to 40% B over 20 minutes, and was equilibrated for 35 minutes at 40% B.

### Immunohistochemical staining

Excised tumor tissues from all animal cohorts were immersion fixed in 4% paraformaldehyde (Ted Pella Inc., Redding, CA) in a 0.1 M phosphate buffer (pH 7.4) at room temperature for 2 hours and then transferred to 4 °C overnight. Twenty-four hours later, tissues were transferred to 30% sucrose in a 0.1 M phosphate buffer for cryoprotection for 48 hours. Tissues were then embedded in OCT (Sakura Finetek USA Inc., Torrance, CA; Cat# 4583) and frozen using a liquid nitrogen chilled isopentane bath. Cryostat sections (16 µM thick) were then thaw-mounted on slides, dried, and stored at −20 °C. For immunoprocessing, slides were thawed, immersed in 4% paraformaldehyde for 3 minutes (to firmly fix the sections to the slide), and then rinsed in PBS three times for 10 minutes each. Following a 30 minute wash in 5% normal donkey serum (NDS; Jackson ImmunoResearch Laboratories Inc., West Grove, PA; Cat# 017-000-121, RRID: AB_2337258) in PBS (to block non-specific labeling), the slides were incubated for 24–48 hours at 4 °C in rabbit anti-Ki67 antibody (1:500) (Abcam, Cambridge, MA; Cat# ab16667, RRID: AB_302459) diluted in PBS containing 0.3% Triton-X 100 and 5% NDS. Slides were then washed with PBS and incubated for 1 hour at room temperature with biotinylated donkey anti-rabbit antibody (1:500) (Jackson ImmunoResearch Laboratories Inc., West Grove, PA; Cat# 711-065-152, RRID: AB_2340593) diluted in PBS with 0.3% Triton X-100 and 5% NDS. Next, slides were washed with PBS, incubated in ImmPACT DAB (Vector Laboratories Inc., Burlingame, CA; Cat# SK-4105) for 2 minutes, rinsed with PBS, and air-dried. Slides were counterstained with hematoxylin and eosin. Images were captured using an Infinity 2 color digital camera, and INFINITY software (Teledyne Lumenera, Ottawa, ON). Images were only adjusted for brightness and contrast.

### Quantification and statistical analysis

All data were obtained from greater than/equal to three independent experiments and the n values described in each figure legend represent each independent trial or animal. Data for all experiments were analyzed using a Mann-Whitney *U* test, one-way ANOVA with Tukey post-hoc test, or repeated measures one-way ANOVA with Bonferroni post-hoc test using Prism software (GraphPad Prism, version 8.0.2). All data are represented as means ± SEM and p < 0.05 was considered statistically significant and the degree of significance is indicated in each figure legend.

### Method guidelines

All experiments were performed in accordance with relevant guidelines and regulations, including all experimentation using recombinant or synthetic nucleic acid molecules as approved by the Institutional Biosafety Committee of Stony Brook University (#850844).

### Ethics approval and consent to participate

The animal experiments conducted were approved by the Stony Brook University Institutional Animal Care and Use Committee (#850980).

## Supplementary information

Supplementary Information

## Data Availability

The datasets generated and analyzed during the current study are available from the corresponding author on reasonable request.
