# Supplementary material for: FABP5 coordinates lipid signaling that promotes prostate cancer metastasis
Source: Sci Rep. 2019 Dec 12;9:18944. doi: 10.1038/s41598-019-55418-x (PMC6908725; doi:10.1038/s41598-019-55418-x)

Supplementary information

FABP5 coordinates lipid signaling that promotes prostate cancer metastasis

Gregory Carbonetti[1,2,3], Tessa Wilpshaar[1,2], Jessie Kroonen[1,2], Keith Studholme[2], Cynthia Converso[1,3], Simon d'Oelsnitz[1], and Martin Kaczocha[1,2,4*]

[1]Department of Biochemistry and Cell Biology, Stony Brook University, Stony Brook, NY, 11794, USA

[2]Department of Anesthesiology, Stony Brook University, Stony Brook, NY, 11794, USA

[3]Graduate Program in Molecular and Cellular Biology, Stony Brook University, Stony Brook, NY, 11794, USA

[4]Institute of Chemical Biology and Drug Discovery, Stony Brook University, Stony Brook, NY, 11794, USA

* Corresponding author: correspondence and requests for materials should be addressed to MK (Martin.Kaczocha@Stonybrook.edu).

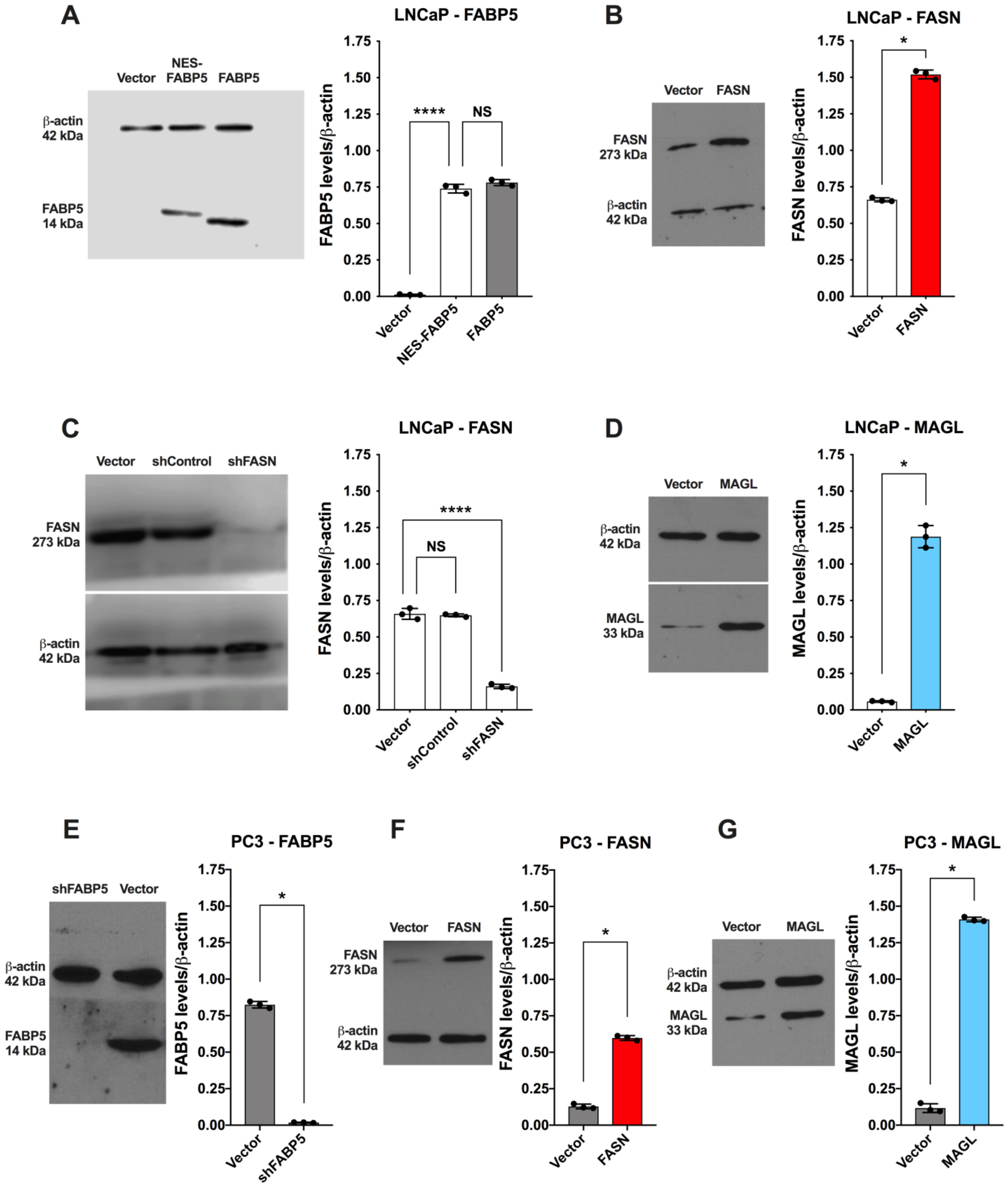

Supplementary Figure S1. Protein expression in virally-transduced LNCaP and PC3 cells.
(A-D) Representative western blots of FABP5, FASN, and MAGL expression in LNCaP cells following lentiviral infection. FABP5, FASN, and MAGL expression in LNCaP cells were quantified by densitometry analysis of western blots. The signals were normalized to β-actin. In both panels C and D, samples were derived from the same experiment and blots were processed in parallel. (E-G) Representative western blots of FABP5, FASN, and MAGL expression in PC3 cells following lentiviral infection. FABP5, FASN, and MAGL expression levels in PC3 cells were quantified by densitometry analysis of western blots. The signals were normalized to β-actin. Data are presented as means ± SEM. *, $p < 0.05$; ****, $p < 0.0001$; NS, not significant; ($n = 3$).

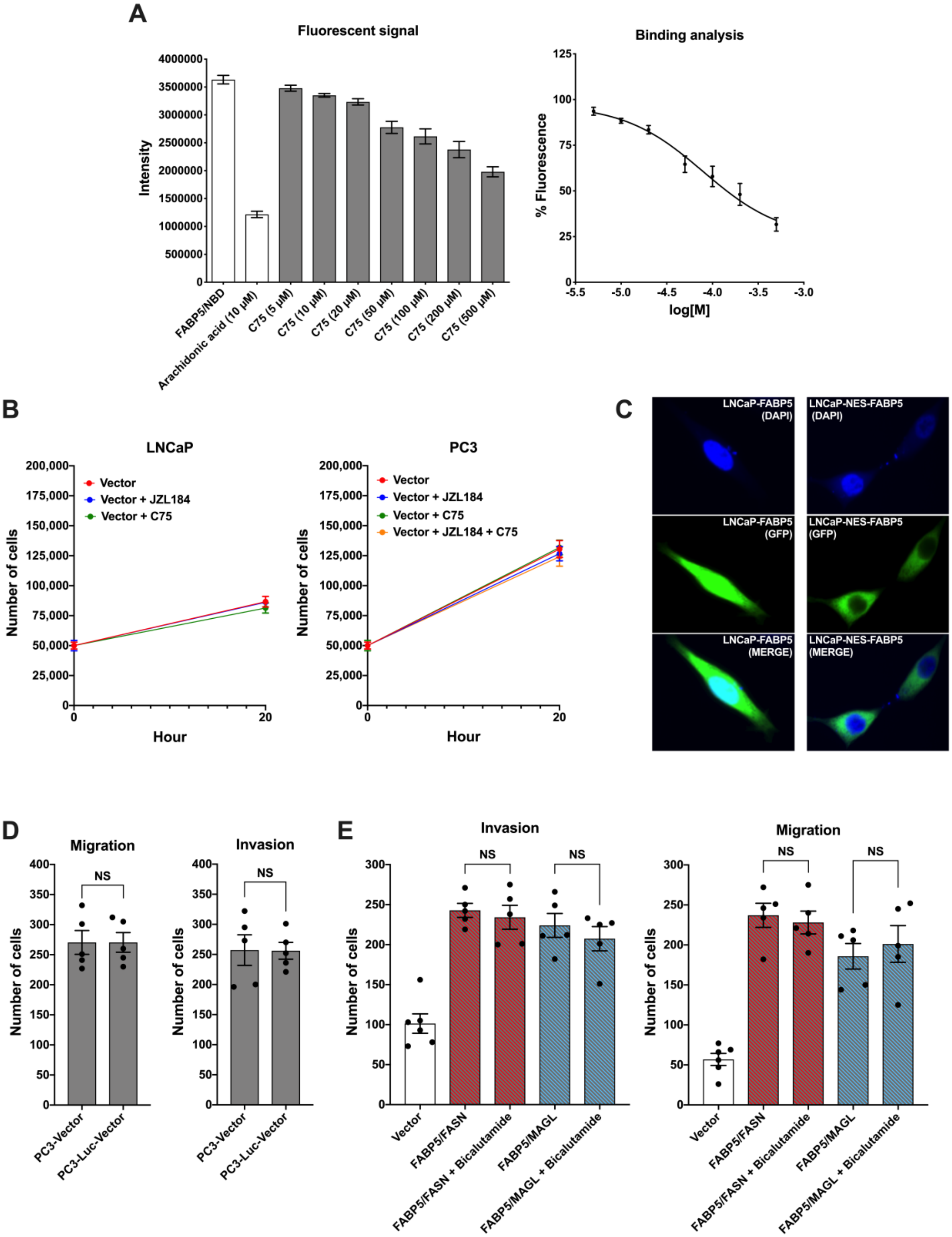

Supplementary Figure S2. Characterization of cell-lines and role of androgen receptors in FASN- and MAGL-mediated metastatic potential.

(A) Binding of C75 to FABP5. Displacement of the fluorescent NBD-stearate (0.5 µM) from purified FABP5 by arachidonic acid (10 µM, positive control) and increasing concentrations of C75 (5-500 µM) (n = 3). (B) Proliferation of vector-expressing LNCaP or PC3 cells over 20 hours in the presence of JZL184 (10 µM) or C75 (40 µM) or JZL184 and C75. Data are presented as means ± SEM (n = 3). (C) Localization of FABP5 in FABP5- and NES-FABP5 expressing LNCaP cells. Green: GFP-tagged FABP5 or NES-FABP5; blue: DAPI. (D) Migration and invasion of PC3-Vector and PC3-Luc-Vector cells *in vitro*. Data are presented as means ± SEM. NS, not significant; (n = 5). (E) Migration and invasion of LNCaP cells co-expressing FABP5 with FASN or MAGL upon treatment with vehicle or the androgen-receptor antagonist bicalutamide (10 µM). Data are presented as means ± SEM. NS, not significant; (n = 5).

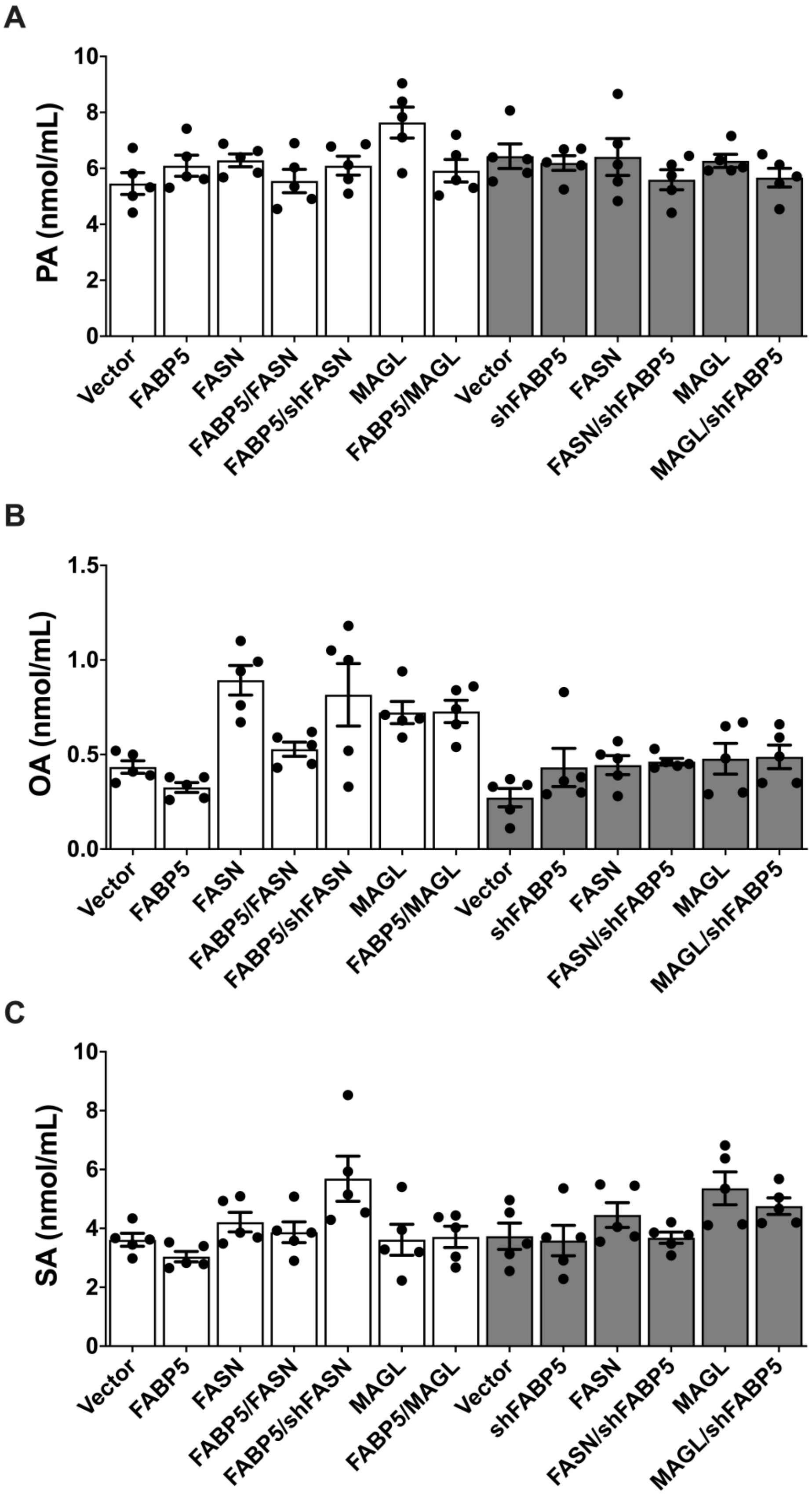

Supplementary Figure S3. Free fatty acid levels in LNCaP and PC3 cells.
Levels of palmitic acid (A), oleic acid (B), and stearic acid (C) in the virally-transduced LNCaP (white bars) and PC3 (grey bars) cell-lines. Data are presented as means ± SEM (n = 5).

Additional Supporting Materials
Original Images for Western Blots in Figure 5 and Supplementary Figure S1

Original Blot – Figure 5B (Top Panel):

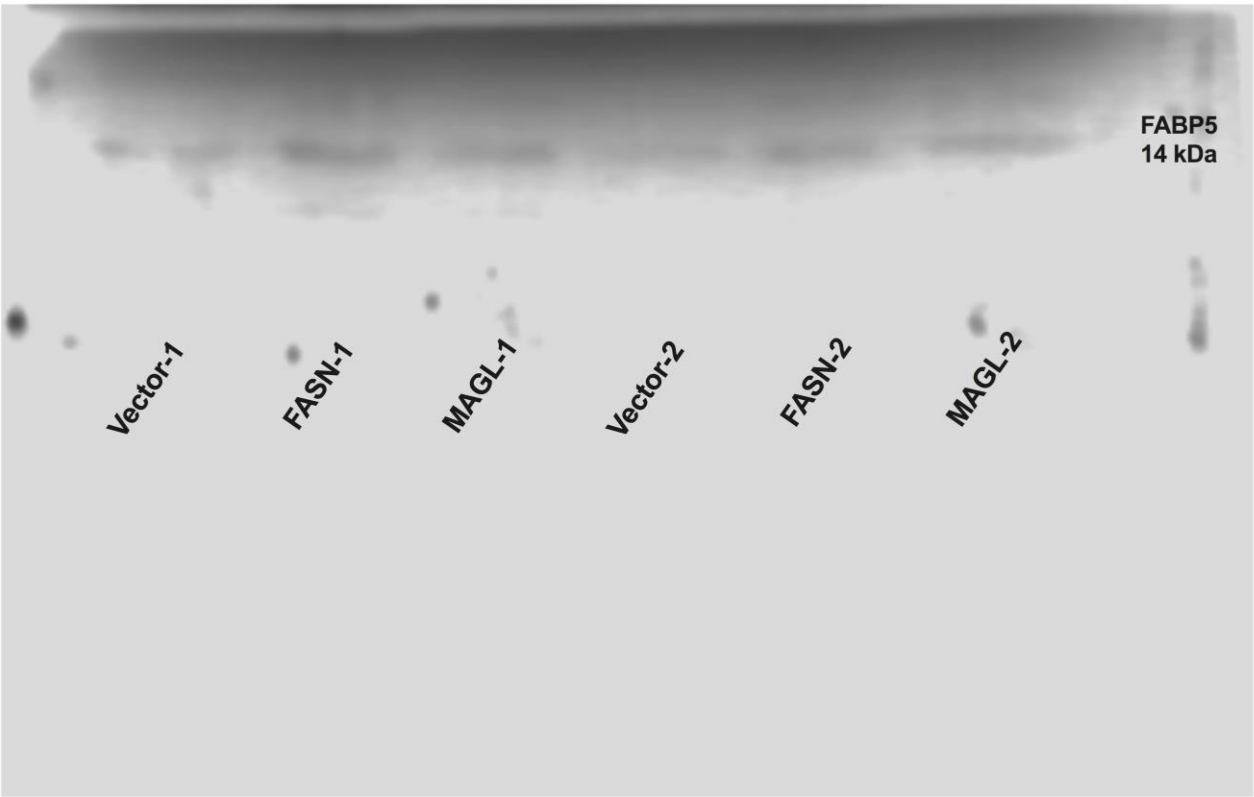

Original Blot – Figure 5B (Bottom Panel):

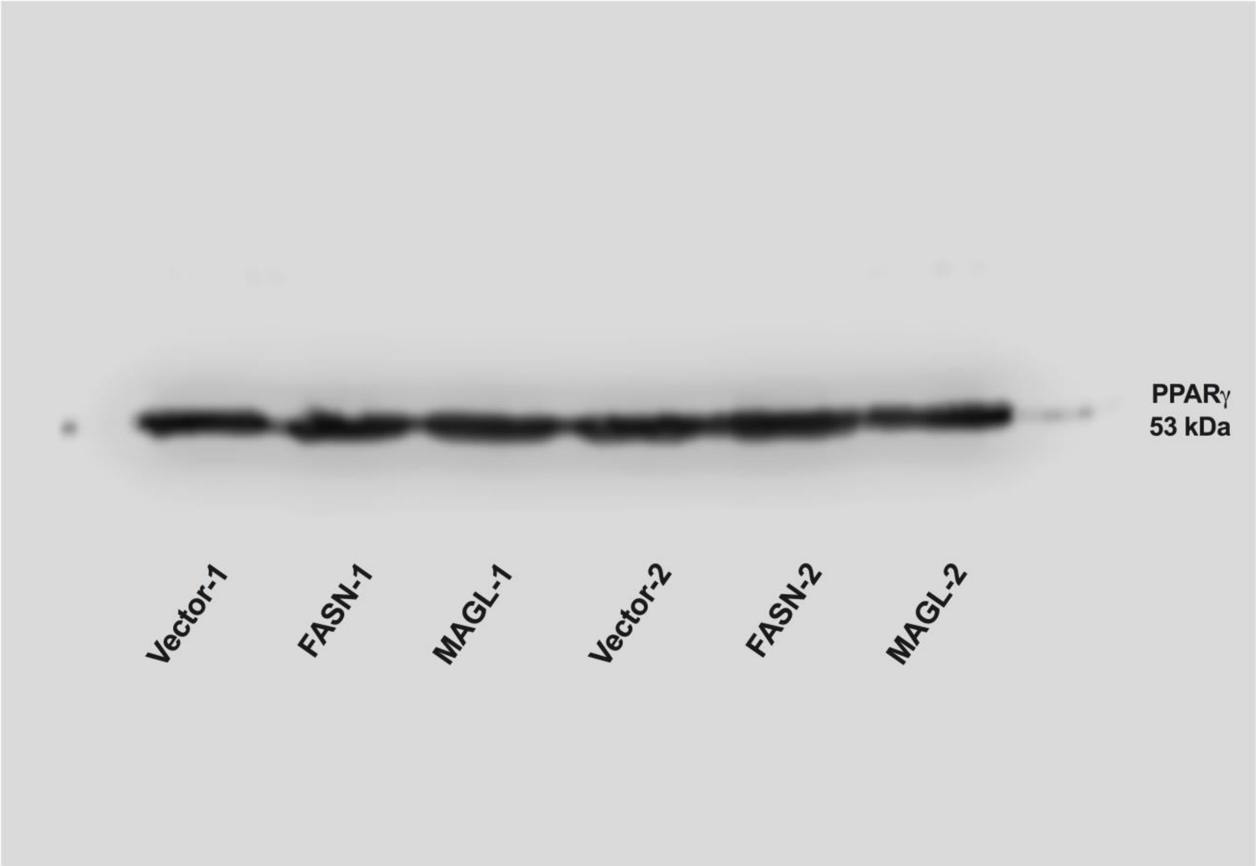

Original Blots – Figure 5B (Right Panel):
Top, Low exposure blot in which signals were not observed.
Bottom, High exposure blot in which only non-specific signals were observed.

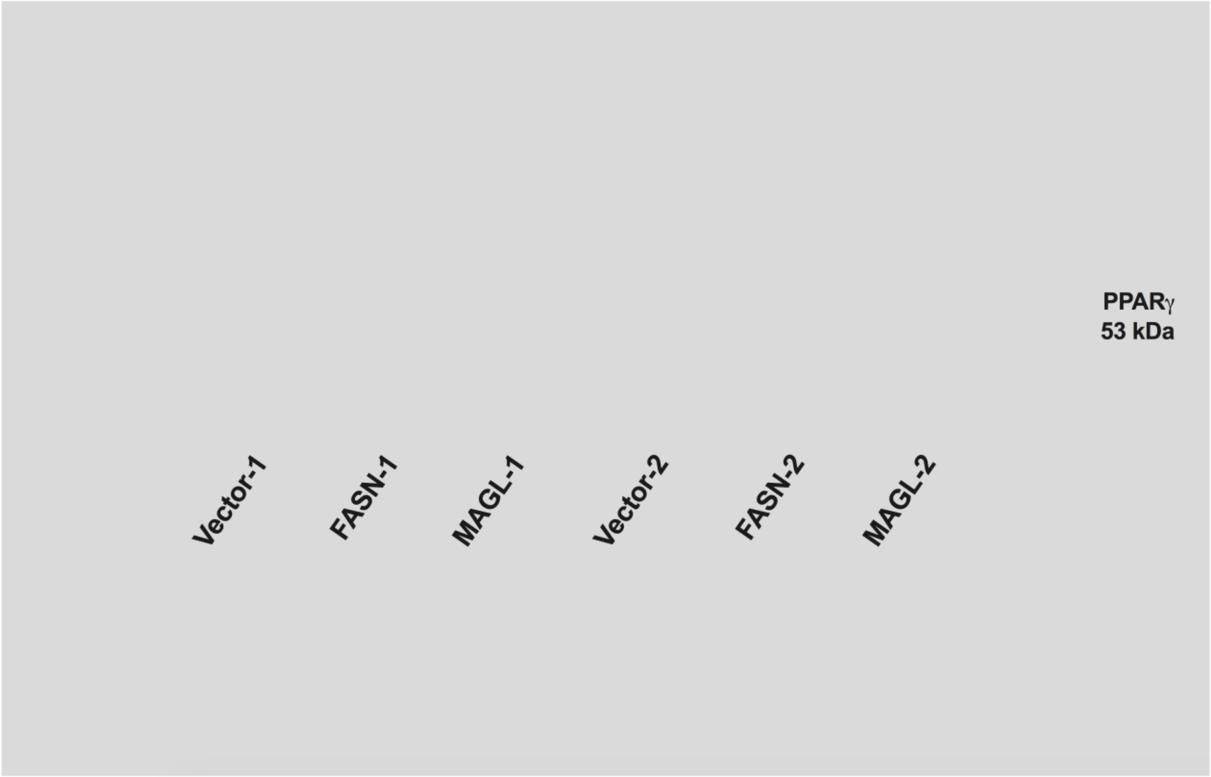

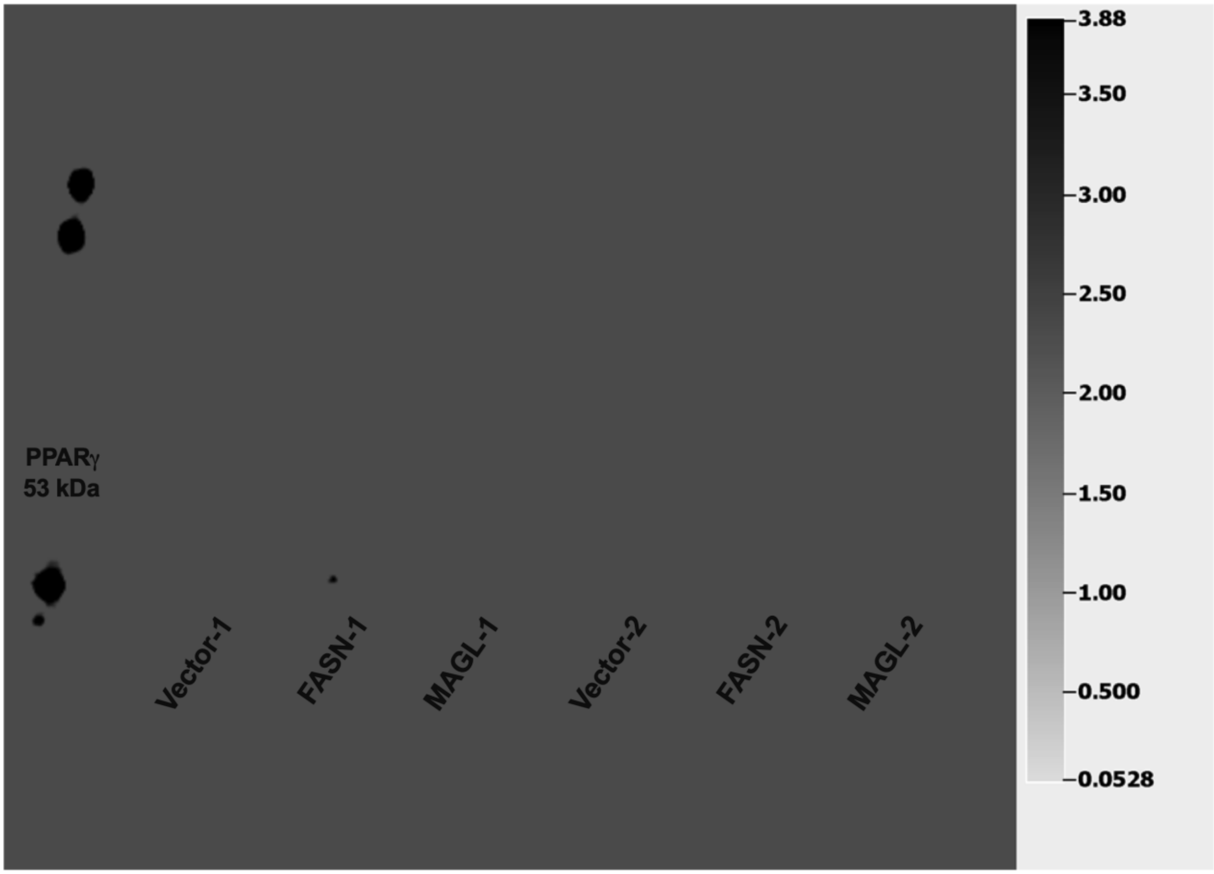

Original Blot – Figure 5D (Top Panel):

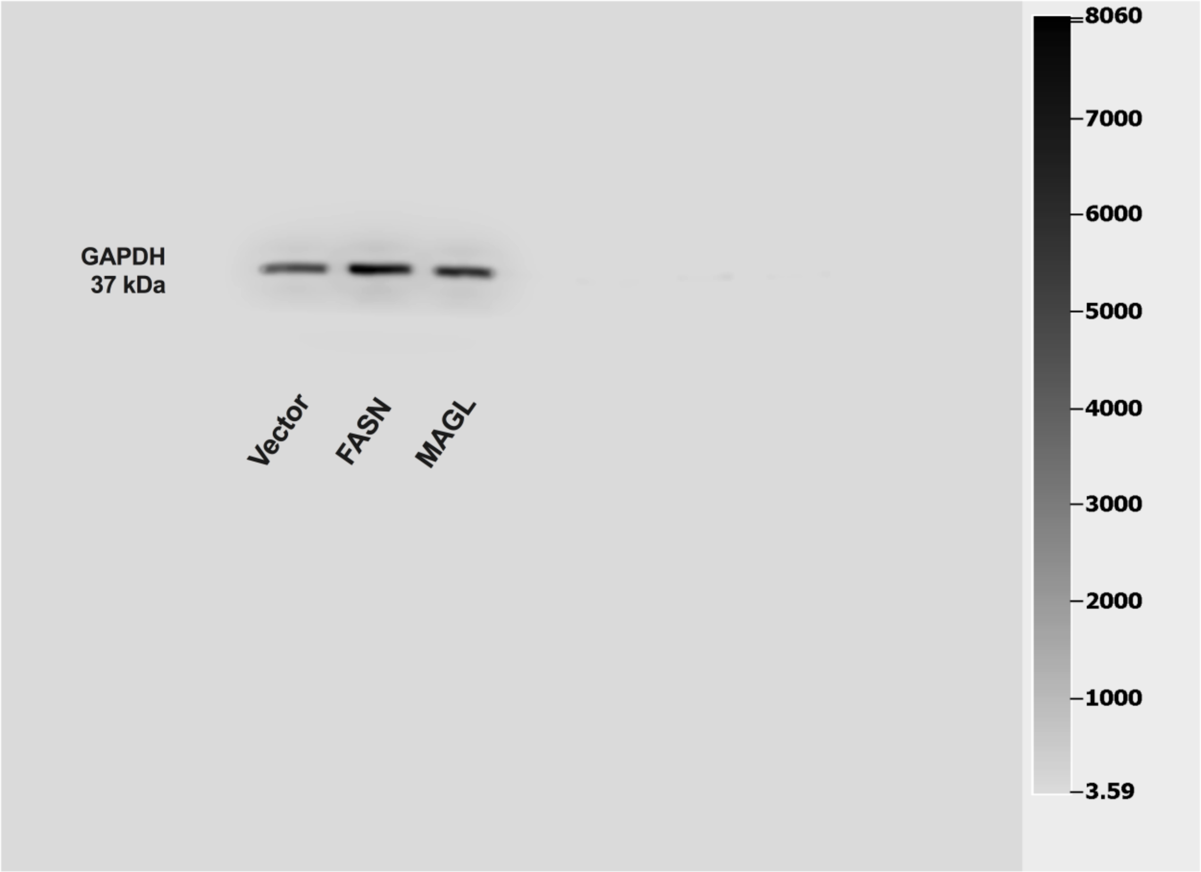

Original Blot – Figure 5D (Bottom Panel):

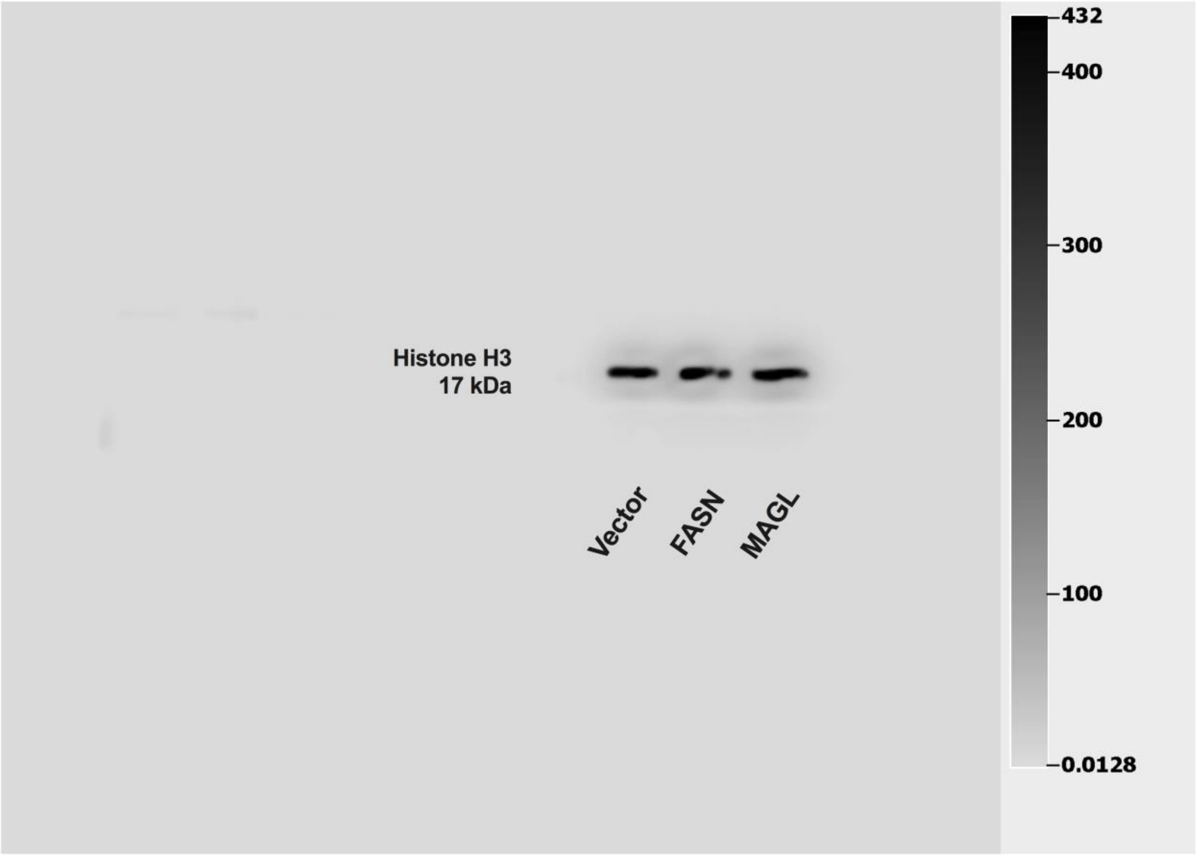

Original Blot – Figure 5E:

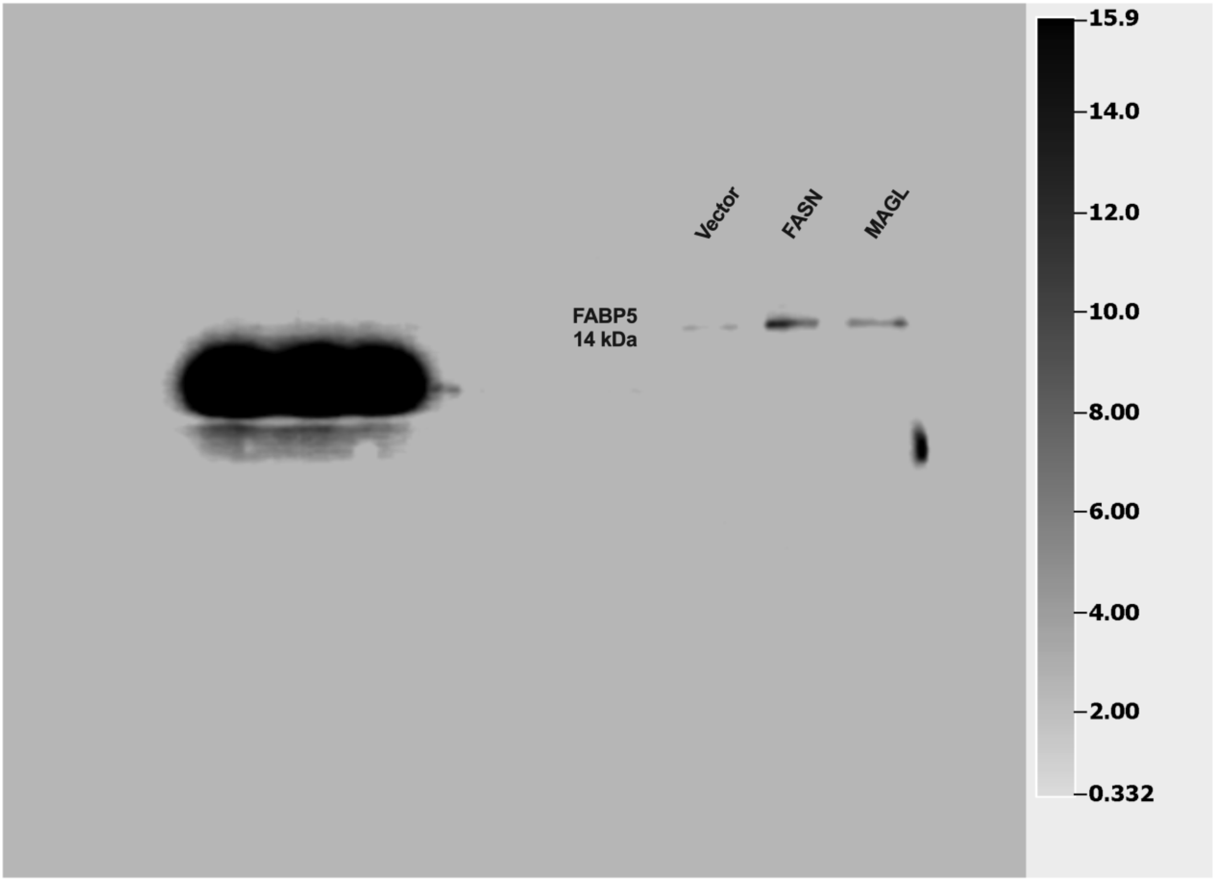

Original Blot – Supplementary Figure S1A:

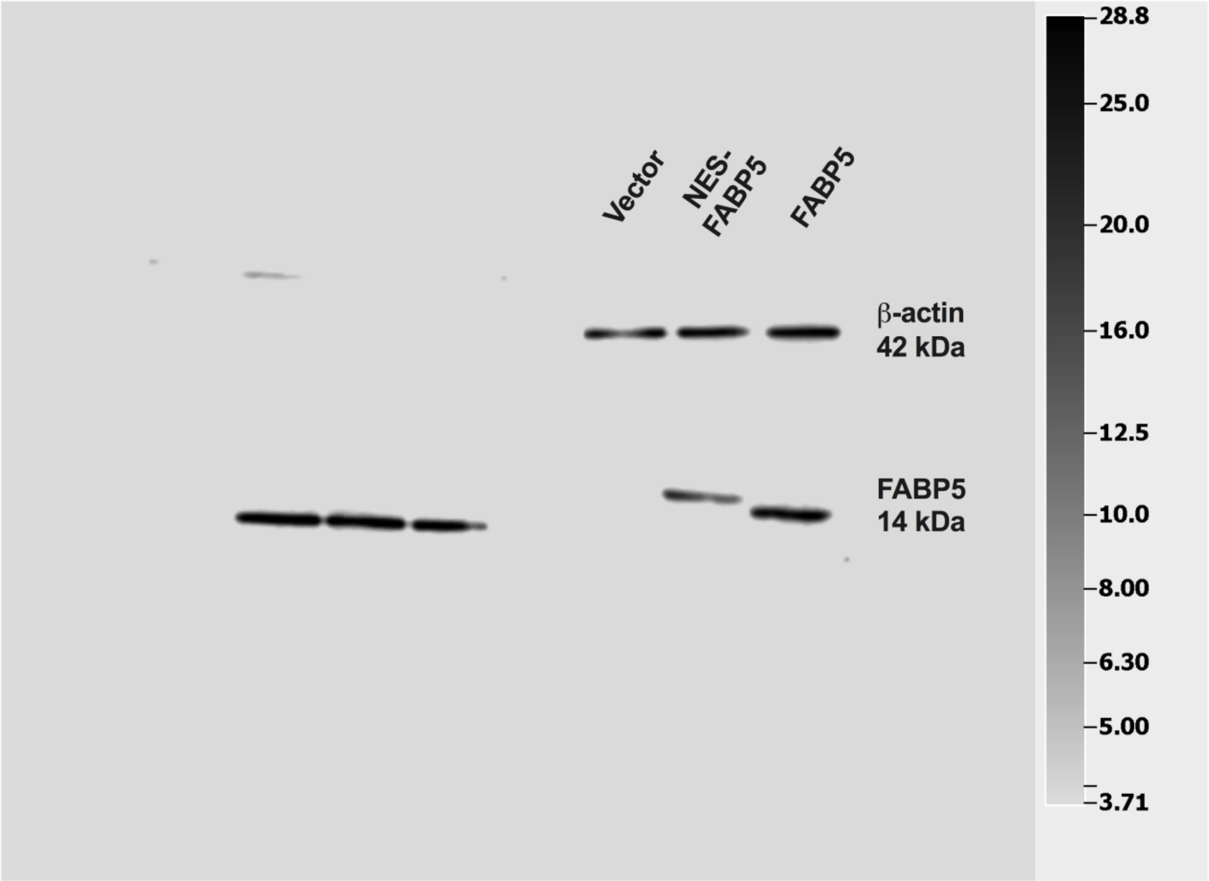

Original Blot – Supplementary Figure S1B:

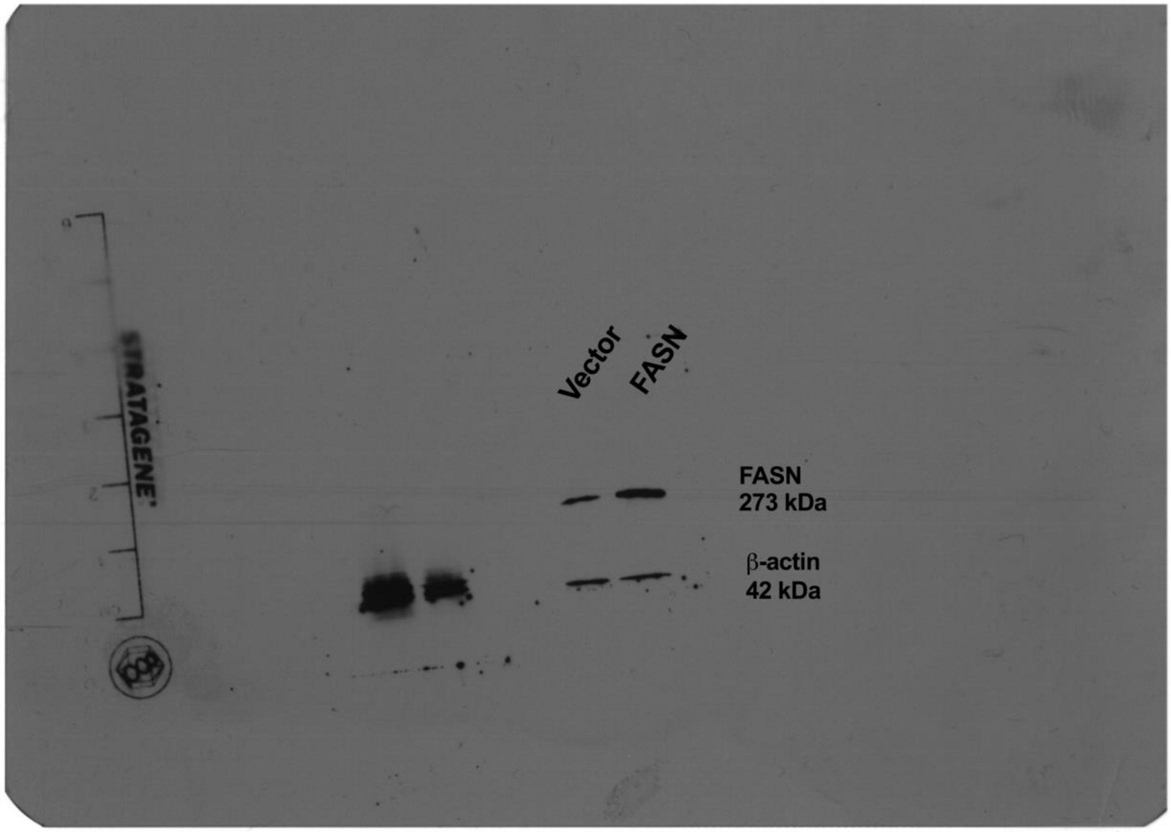

Original Blot – Supplementary Figure S1C (Top Panel):

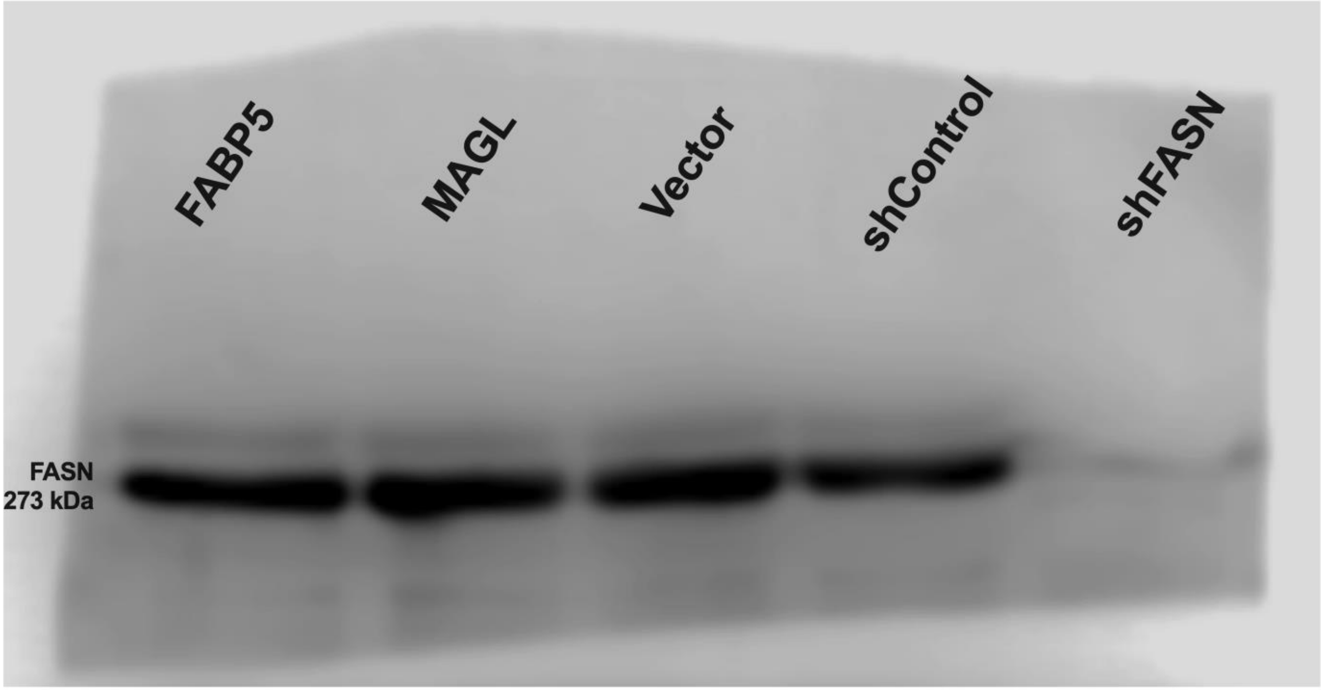

Original Blot – Supplementary Figure S1C (Bottom Panel):

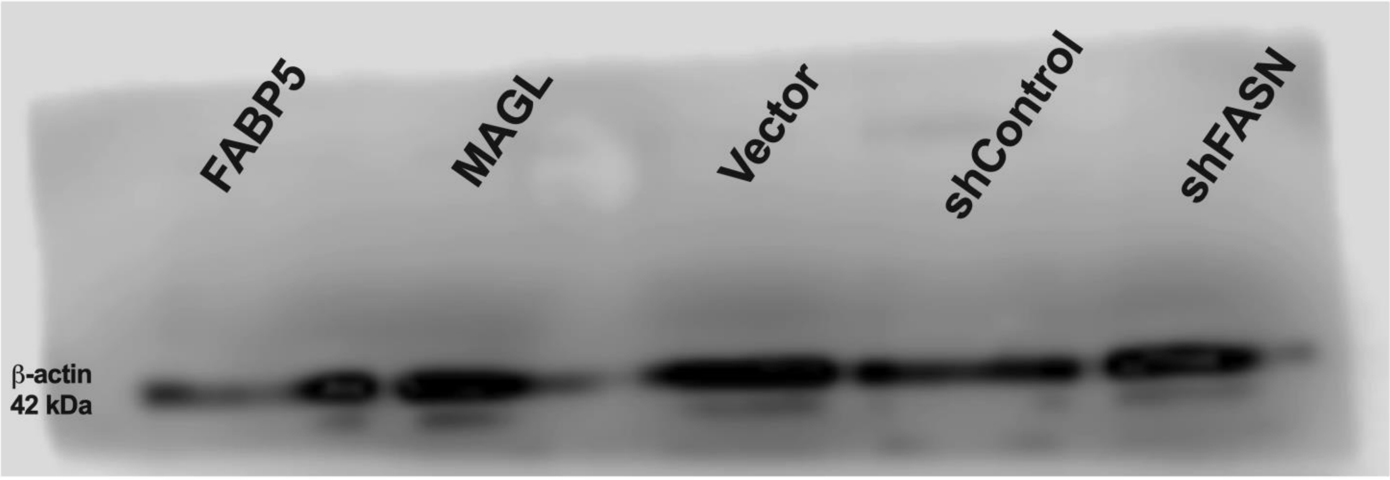

Original Blot – Supplementary Figure S1D:

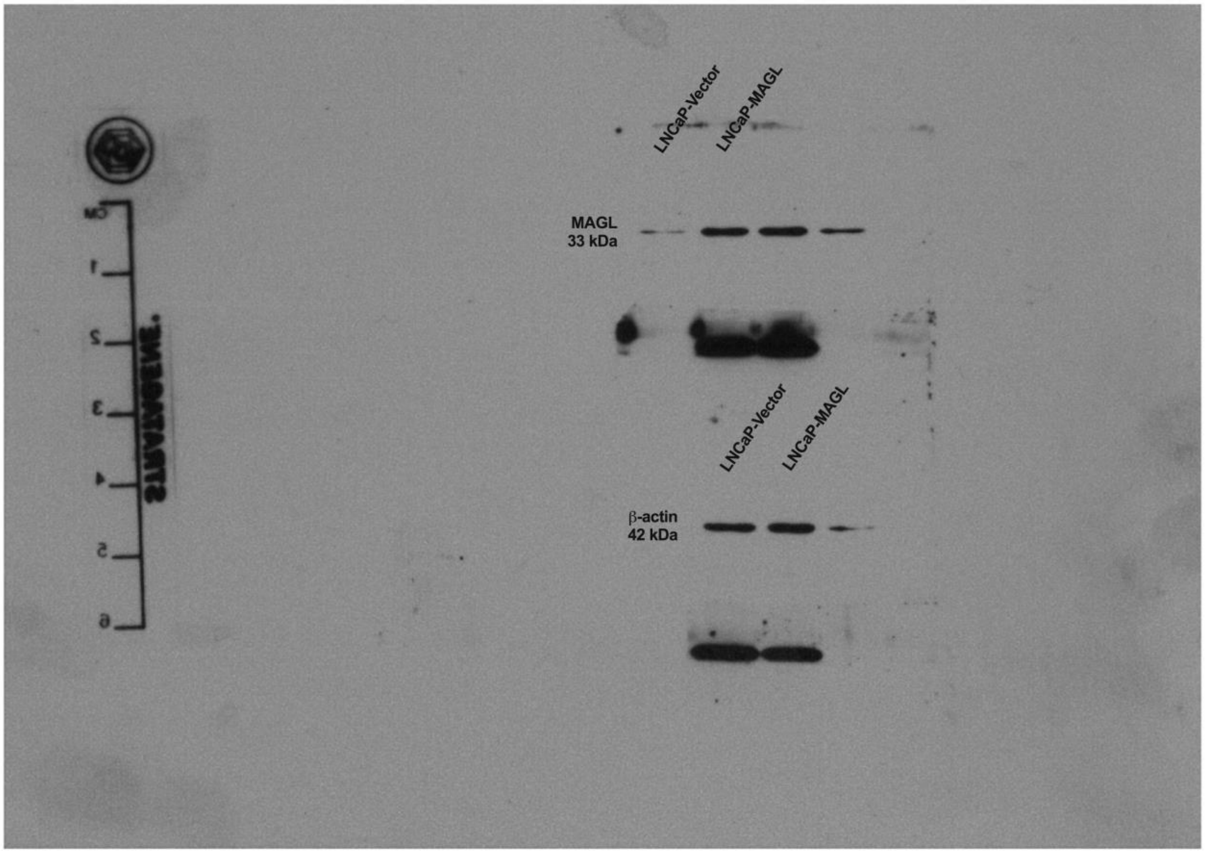

Original Blot – Supplementary Figure S1E:

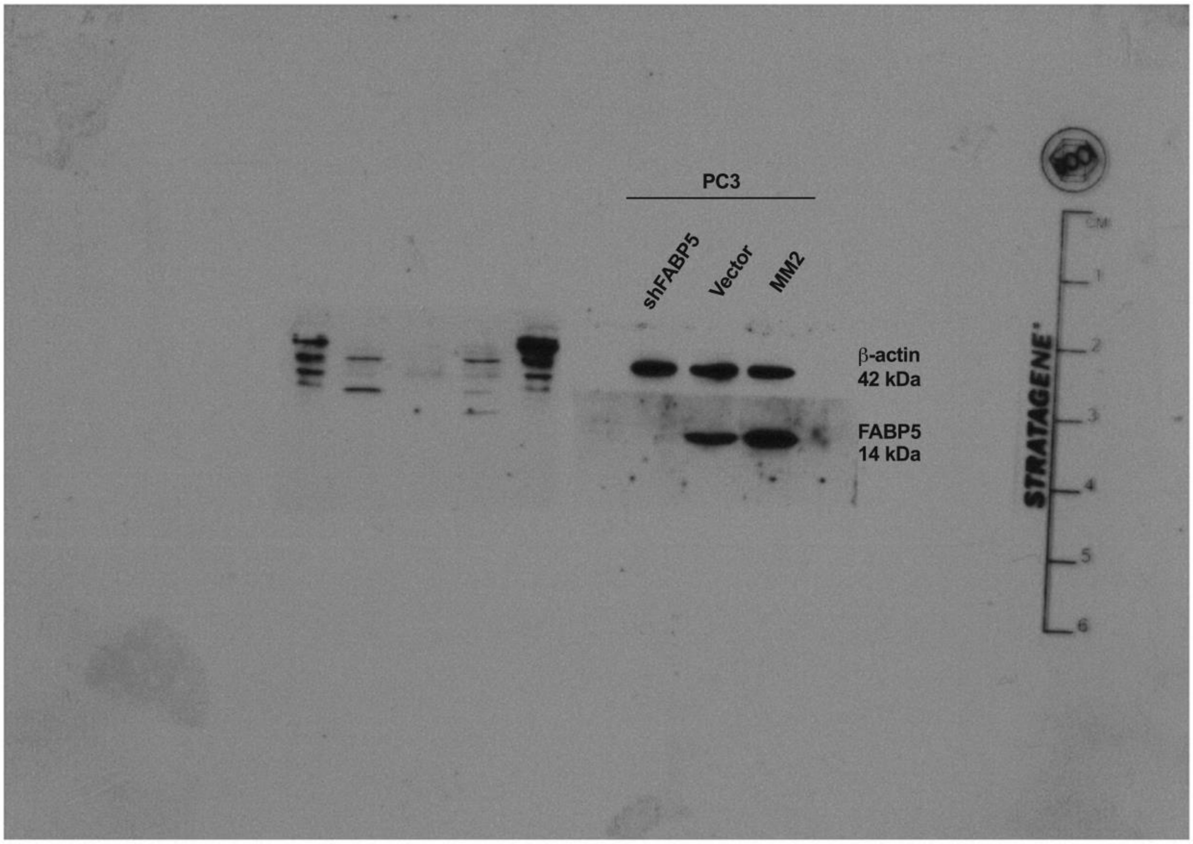

Original Blot – Supplementary Figure S1F:

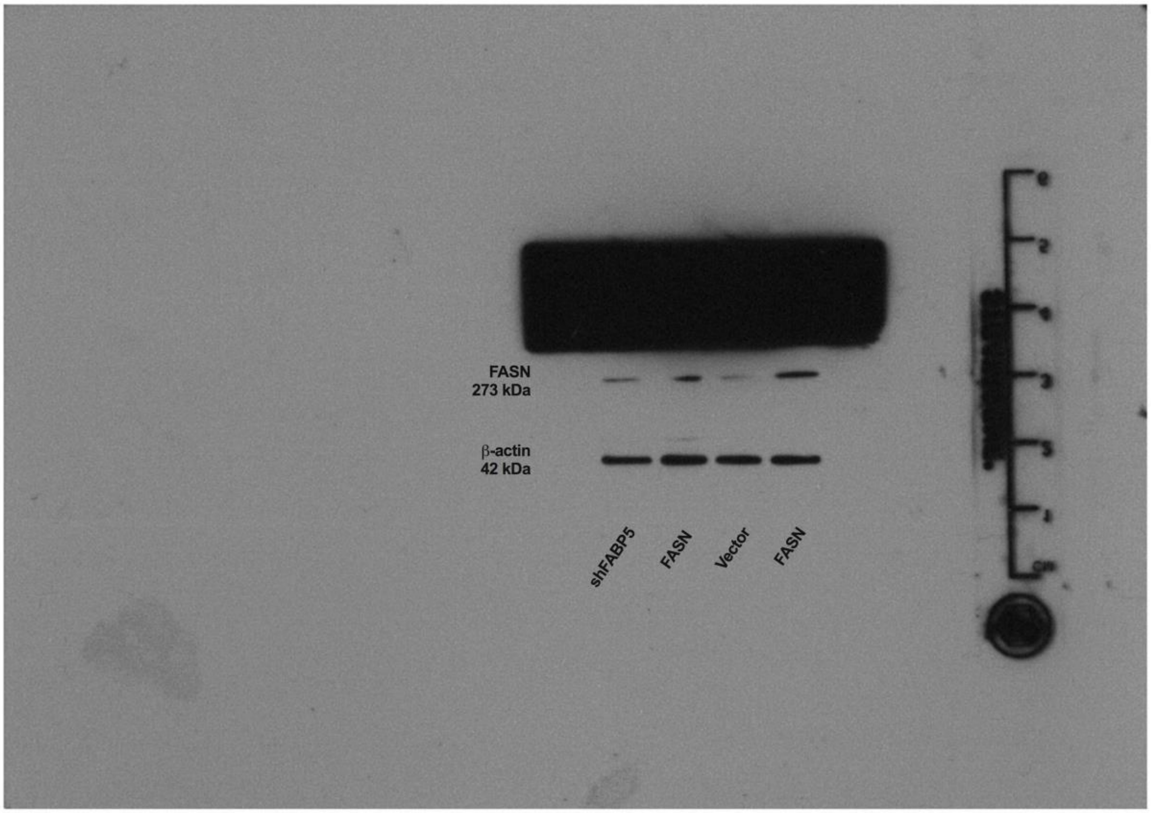

Original Blot – Supplementary Figure S1G:

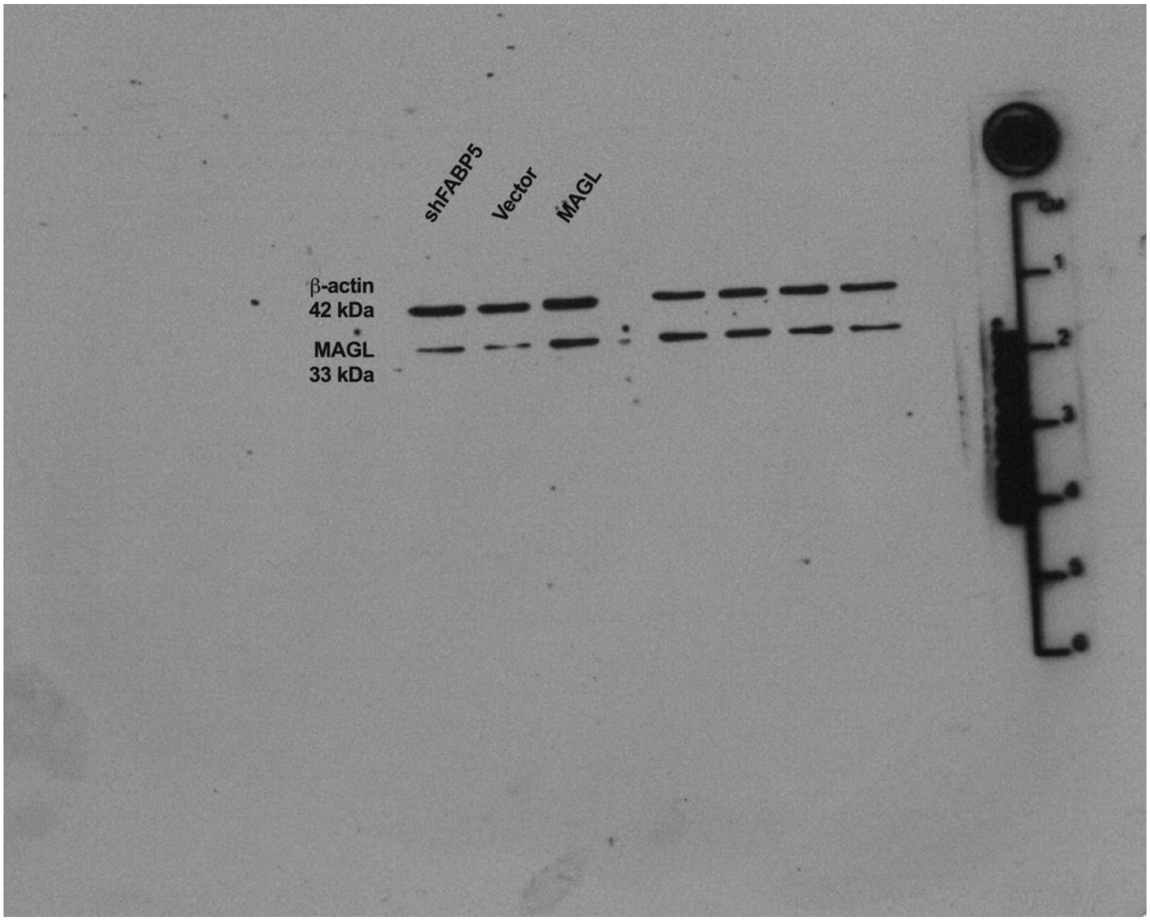

Supplement: Supplementary file 1 — Supplementary Information [file 41598_2019_55418_MOESM1_ESM.pdf]
